# Probiotics, Prebiotics, and Synbiotics as Oral Supplements for Skin Health, Function, and Disease Throughout the Life Course: A Scoping Review

**DOI:** 10.1093/nutrit/nuaf205

**Published:** 2025-11-29

**Authors:** Rebecca A Hillier, Rachel Gibson, Thivi Maruthappu, Kevin Whelan, Emily J Prpa, Holly R Neill, Charlotte G Phillips, Wendy L Hall

**Affiliations:** Department of Nutritional Sciences, School of Life Course and Population Sciences, King’s College London, London, SE1 9NH, United Kingdom; Department of Nutritional Sciences, School of Life Course and Population Sciences, King’s College London, London, SE1 9NH, United Kingdom; Department of Nutritional Sciences, School of Life Course and Population Sciences, King’s College London, London, SE1 9NH, United Kingdom; Department of Nutritional Sciences, School of Life Course and Population Sciences, King’s College London, London, SE1 9NH, United Kingdom; Department of Nutritional Sciences, School of Life Course and Population Sciences, King’s College London, London, SE1 9NH, United Kingdom; Yakult UK & Ireland Limited, South Ruislip, Middlesex, HA4 6QQ, United Kingdom; Department of Nutritional Sciences, School of Life Course and Population Sciences, King’s College London, London, SE1 9NH, United Kingdom; Yakult UK & Ireland Limited, South Ruislip, Middlesex, HA4 6QQ, United Kingdom; Yakult UK & Ireland Limited, South Ruislip, Middlesex, HA4 6QQ, United Kingdom; Department of Nutritional Sciences, School of Life Course and Population Sciences, King’s College London, London, SE1 9NH, United Kingdom

**Keywords:** dermatology, microbiome, prebiotics, probiotics, skin, synbiotics

## Abstract

In this review we sought to map the body of published literature on the role of oral probiotics, prebiotics, and synbiotics in maintaining and optimizing skin health and function and preventing and managing skin conditions throughout the life course. Globally, the burden of skin diseases is considerable. Diet is a modifiable risk factor for many dermatological conditions, and one mechanism by which nutrition influences skin health is through the gut microbiome. Oral probiotics, prebiotics, and synbiotics have the potential to improve skin health, delay skin aging, and successfully treat dermatological diseases. We developed a scoping review protocol in accordance with the Johanna Briggs Institute methodology. Six online databases were systematically searched for peer-reviewed literature, and non–peer-reviewed sources were also considered. All records were screened independently by 2 reviewers using predefined eligibility criteria. A total of 516 studies were included in the scoping review, comprising 73 systematic reviews. Most studies investigated probiotics (*n* = 401). Infants (0-12 months old) and adults (18-60 years old) were the age groups most frequently receiving supplementation with probiotics (42% [*n* = 114] and 41% [*n* = 112] of human studies, respectively), whereas only 15% of studies (*n* = 41) comprised adults participants older than 60 years. Of the skin diseases investigated, atopic dermatitis was the most extensively researched (*n* = 330 studies), followed by psoriasis (*n* = 24), and acne (*n* = 23). Skin health and function in healthy populations is a growing area of research; outcomes related to wrinkling, elasticity, aging, or UV irradiation response accounted for 54 studies. Consistencies in the evidence base found in our investigation underscore the need for an umbrella review on oral probiotics, prebiotics, and synbiotics and atopic dermatitis, as well as a systematic review on skin aging. Preliminary evidence for roles in managing rosacea, alopecia, and melasma suggests additional research avenues. Future studies should consider participant diets, probiotic strain and dose reporting, and inclusivity of populations and languages.

## INTRODUCTION

From infancy to old age, skin health and function undergo numerous changes, while some skin conditions confer a greater burden at specific life stages. For example, atopic dermatitis in children, acne in adolescents, psoriasis in individuals aged 45-75, and ulcers in adults older than 75 years. Meanwhile, other skin conditions, such as dermatological cancers, increase in prevalence throughout the life course.^1^ Skin aging and its consequences, such as dry skin, itching, infections, and tumors, have been identified as one of the most critical challenges in global skin health.^2^ Across the global population, the burden of skin diseases is considerable, representing the fourth most significant cause of disability, excluding mortality.^1^ The maintenance and optimization of skin health are known to be affected by both endogenous factors, such as genetics, and exogenous factors, including sun radiation, pollution, smoking, and nutrition.^3^ One of the mechanisms by which nutrition influences skin health is via the gut microbiome. The concept of a gut–skin axis was first posited in 1930 but has received increasing attention over the last decade, with researchers suggesting a range of mechanisms by which the gut microbiome affects skin and potentially vice versa.^4^^,^^5^

Probiotics (live microorganisms that, when administered in adequate amounts, confer health benefits). When probiotics are delivered orally, their health benefits are most commonly elicited through modulation of the gut microbiome.^6^^,^^7^ Similarly, oral prebiotics (substrates selectively utilized by host microorganisms and confer a health benefit) and synbiotics (mixtures comprising live microorganisms and substrates selectively utilized by host microorganisms to confer a health benefit) also modulate the gut microbiome.^8^^,^^9^ This ability to modulate the gut microbiome suggests that oral probiotics, prebiotics, and synbiotics may also have the potential to improve functional indicators of skin health, delay skin aging, and treat dermatological diseases.

The link between oral probiotics, prebiotics, and synbiotics and skin health has been well researched in certain areas. Our preliminary searches revealed that systematic reviews had been conducted on the effects of oral probiotics in the prevention and treatment of specific skin conditions, particularly atopic dermatitis,^10–13^ whereas for other areas of skin health, the extent of the evidence was less clear. To our knowledge at the time of this report, no comprehensive mapping of the available literature had been performed across all 3 potential therapies—oral probiotics, prebiotics, and synbiotics—and the full range of skin health and disease outcomes throughout the life course. Given the breadth of the concept, which encompasses all aspects of skin health, and the known heterogeneity of the literature regarding probiotics and prebiotics, we elected to perform a scoping review to facilitate a rigorous, comprehensive, and systematic mapping of all available literature. This approach is particularly suitable for identifying areas of research saturation and knowledge gaps. By including all age groups, oral intervention types (probiotics, prebiotics, synbiotics), and cosmetic, functional, and clinical skin-related outcomes in this review, we sought to reflect the potential of modulating the gut–skin axis across the life course. The resulting evidence map is intended to support prioritization of targeted research in both clinical and nonclinical contexts. We hoped to provide the probiotics, prebiotics, and synbiotics industry with a clear indication of the type and quantity of evidence available, including areas for which evidence is sufficient to guide health claim dossiers and those for which further research could open possibilities for new product development.

This scoping review was conducted in accordance with an a priori registered protocol^14^ and the Joanna Briggs Institute (JBI) methodology for scoping reviews,^15^ including the PCC (population, concept, context) framework for the search strategy.

We aimed to map the existing evidence on the use of oral probiotics, prebiotics, and synbiotics for skin-related outcomes across the life course. The objectives were the following:

- Describe all types of evidence available across a range of indicators of skin health and function and skin aging, as well as outcomes related to the prevention and management of skin conditions
- Chart evidence by types of oral probiotics, prebiotics, and synbiotics investigated; by categories of outcomes; and by ethnicity and life stages
- Identify gaps in the literature and make recommendations for research priorities

The research question was as follows: in humans and animals (population), what evidence is available on the effects of oral probiotics, prebiotics, and synbiotics on maintaining and optimizing skin health and function, and preventing or managing skin conditions (concept) across all study settings relevant to oral delivery (context)?

## METHODS

### Search Strategy

Our search strategy was aimed at locating both peer-reviewed (up to March 2024) and non–peer-reviewed (up to June 2024) published or unpublished studies. An initial limited search of MEDLINE was undertaken to identify reports on the topic. The text words contained in the titles and abstracts of relevant reports, along with the index terms used to describe them, were used to develop a comprehensive search strategy for MEDLINE. This comprehensive search strategy, including all identified keywords and index terms, was then adapted for the following databases: EMBASE, CENTRAL, CINAHL, Scopus, and Web of Science. Support in developing and adapting the search strategy was provided by a Senior Library Assistant at King’s College London. Full search strategies for each of these 6 databases are presented in Supplementary Materials 1. To ensure complete evidence mapping, no limits were applied based on language or date of publication. Where necessary, Google Translate or DeepL was used to screen and extract data from studies written in non-English languages.^16^

Sources of non–peer-reviewed literature were also searched using similarly adapted search strategies. These included clinical trial registers (specifically the WHO International Clinical Trials Registry Platform Search Portal, the United Kingdom Dermatology Clinical Trials Network, and the Global Resource for Eczema Trials), pre-print servers (utilizing Scopus), and ProQuest Dissertations and Theses Global. Additionally, abstracts available online in May 2024 were manually searched if they were presented during the 5 years before May 2024 at the following conferences: the British Association of Dermatology, Society for Investigative Dermatology, International Scientific Association for Probiotics and Prebiotics (ISAPP), Japanese Dermatology Society, and European Academy of Dermatology and Venereology. Considering the large number of included studies, the screening of reference lists for additional studies was limited only to those of systematic reviews.

### Inclusion Criteria

Sources were considered for inclusion in the scoping review based on criteria established in advance using the participants, concept, and context framework.

Participants included humans and animals, provided the skin outcomes were relevant to humans (for example, excluding those related to skin mucus in fish or foot pad dermatitis in chickens), with no exclusion criteria based on any participant characteristics. Studies that included participants with a minimum age of 18 years were classified as adult studies. In contrast, studies that included participants aged 0-19 years were used to define pediatric populations (infants, younger children, and older children). This approach enabled us to accurately capture studies relevant to adolescents while maintaining a clear distinction between pediatric and adult populations.

The concept of interest was exposure to oral probiotics, prebiotics, or synbiotics, and measurement of outcomes related to skin health, function, or disease. Concerning exposure, evidence was included if it was derived from investigation of the effects of oral probiotics, prebiotics, or synbiotics using any strain or combination of strains, dose, and duration. Probiotics and synbiotics were defined according to the definitions previously stated by the ISAPP. The ISAPP prebiotic definition includes possible prebiotic candidates such as polyphenols, which are widely studied for their health benefits beyond their prebiotic effect on gut microbiome modulation. Prebiotics were therefore defined in this review as follows: (1) inulin-type fructans (ITFs) or galactooligosaccharides (GOSs); (2) any compound defined by the author as a prebiotic if justification for the compound fulfilling criteria as a prebiotic was explicitly stated (eg, prebiotic xylo-oligosaccharides);^17^ or (3) substances that could meet ISAPP prebiotic criteria (eg, polyphenols) that demonstrated effects on bifidobacteria and/or lactobacilli in the study. Assessment of the fulfillment of prebiotic criteria (2) and (3) was undertaken by the 2 reviewers, in addition to a third reviewer (K.W.), who is an expert in prebiotic interventions. Oral probiotics, prebiotics, and synbiotics could be presented as powders, capsules, tablets, softgels, or food or beverages. Fermented foods that did not fulfil the criteria for probiotics were excluded. Similarly, postbiotics containing inactivated microbial cells with or without their metabolites were also excluded. Eighteen topical probiotics, prebiotics, and synbiotics that modulate the skin microbiome were excluded due to their contrasting mechanisms of action. Concerning outcomes, evidence was included if outcomes described indicators of skin health or skin function or were related to the prevention or management of skin conditions.

Any human, animal, offspring, or ex vivo studies could be included, and there were no exclusion criteria based on setting, geography, or ethnicity. In vitro studies were excluded because they do not investigate the oral delivery of the exposure of interest.

To ensure breadth of evidence mapping, in this review we considered all experimental, quasi-experimental, and observational study designs for inclusion. Conference abstracts were included only if no corresponding journal article was available. Qualitative studies, commentaries, opinion papers, and nonsystematic reviews were excluded from the analysis. Systematic reviews that met the inclusion criteria were reviewed, and the studies within them were considered against the eligibility criteria for inclusion. Given the high number of systematic reviews available in certain areas, these were also included and analyzed separately to enable comprehensive evidence mapping.

### Study/Source of Evidence Selection

Following the search, all identified citations were collated and uploaded into Covidence systematic review software (Veritas Health Innovation, Melbourne, Australia), and duplicates were removed. After a pilot test of 25 titles and abstracts and a discussion of any disagreements, all titles and abstracts were first screened by R.A.H. and subsequently independently re-screened by any of 3 independent reviewer (either H.R.N., C.G.P., or E.J.P.) against the inclusion criteria. Potentially relevant sources were retrieved in full, and the full texts of selected citations were assessed in detail against the inclusion criteria by 2 independent reviewers from a pool of 3 reviewers (R.A.H., H.R.N., C.G.P.). Any disagreements arising between the reviewers were resolved through discussion, or, where necessary, with the assistance of an additional reviewer (W.L.H., K.W., T.M.).

### Data Extraction

Data extraction was completed between July and October 2024 using Microsoft Excel spreadsheets, which included detailed guidance for each data field to ensure consistency (Supplementary Materials 2). A pilot data extraction of 10 studies was performed independently by 2 reviewers, and any differences were discussed to ensure the consistency of the approach. Subsequent data were extracted by 1 reviewer (either R.A.H. or Y.Q.). As the number of included studies considerably exceeded the originally anticipated number, the decision was made to verify a proportion of the extraction (approximately 15%) by a second reviewer (W.L.H. for those initially extracted by R.A.H. and R.A.H. for those initially extracted by Y.Q.) to balance accuracy and feasibility.^19^ Where necessary, reviewers involved the appropriate author with the relevant expertise to confirm the appropriate classification of the intervention or outcome, respectively. Any disagreements that arose between the reviewers were resolved through discussion or with the assistance of an additional reviewer.

The data extraction template was further developed from the version provided in the protocol to add detail, and 3 additional key modifications were made. First, data from the included systematic reviews were extracted using a tailored data extraction form to ensure the relevance of data fields and facilitate their separate analysis without double-counting the included studies. Second, given the overall high number of included studies, the number of data fields extracted for animal studies was significantly reduced. Third, skin health and function outcomes were only captured in healthy populations to avoid overlap with outcomes related to the management of skin disease. These modifications were deemed appropriate to strike a balance between achieving the review objectives and the time and resources available for the review.

No quality of evidence assessment was made, as this is not required for scoping reviews. Considering the quantity of evidence available, no authors of articles were contacted to request missing or additional data.

### Data Charting

The data charting steps were as follows: (1) A preliminary data charting plan was developed by the review team based on the scoping review protocol. (2) Pilot tables and figures were generated and reviewed by the authors. (3) Linked Excel sheets were used to extract key information to describe overall study characteristics, human experimental study characteristics, and intervention details in tabular form, and to map the evidence by year of publication, skin condition, and life course stage; probiotic, prebiotic, and synbiotic types; frequency of outcomes studied by study type; frequency of demonstration of a beneficial effect; and frequency of demonstration of a beneficial effect by outcomes. (4) Authors iteratively reviewed initial tables and figures. (5) Final versions were approved and agreed upon once final adjustments to interpretation of study data were completed.

## RESULTS

### Study Inclusion

**Figure 1.**
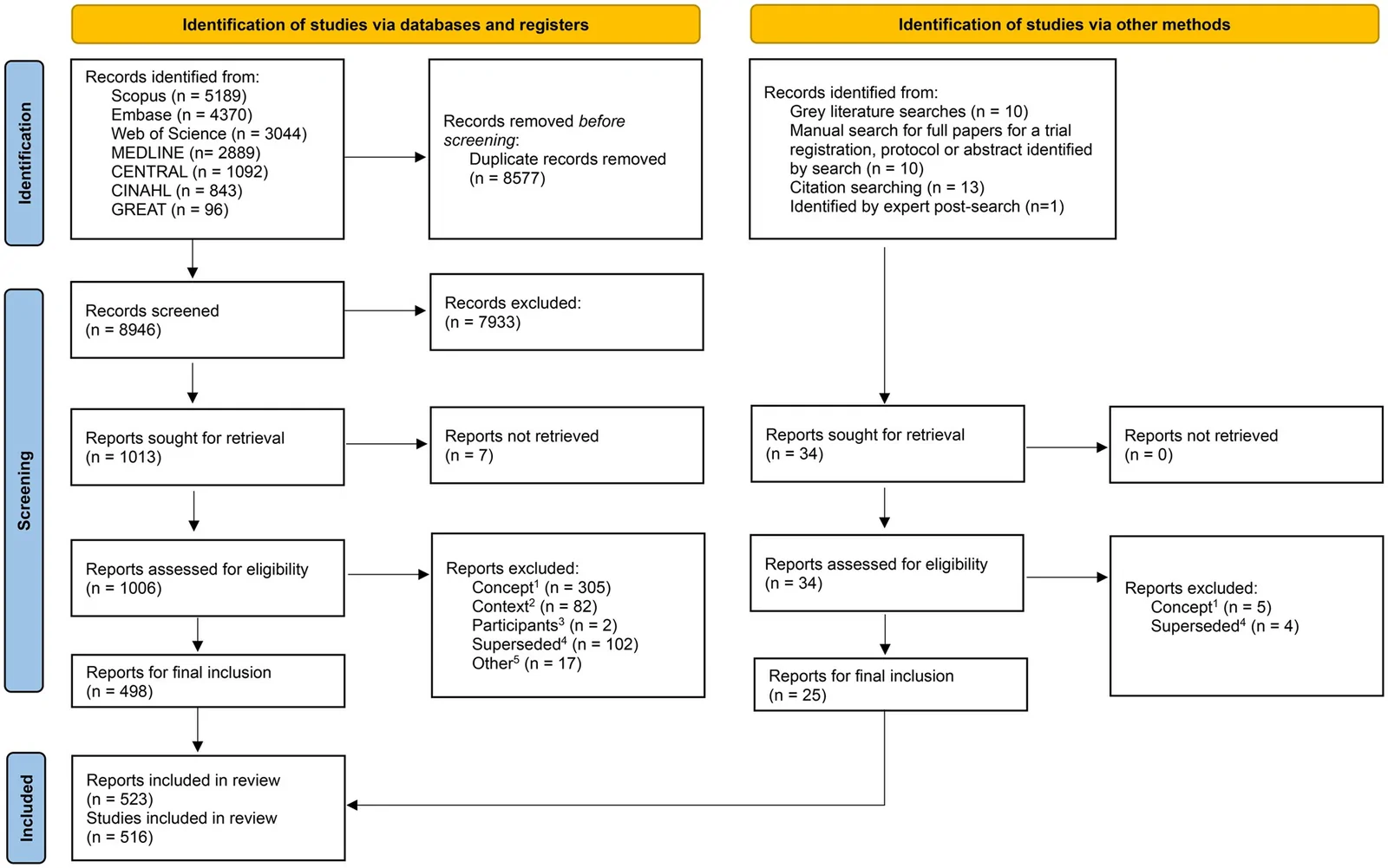
Flow Diagram of Identification, Screening and Selection of Studies ^1^Wrong exposure/intervention (eg, nonoral delivery or postbiotics) or no relevant skin outcome ^2^Wrong experimental design (eg, in vitro) or study type (eg, protocol, trial registration, commentary or non-systematic review) ^3^Irrelevant population (eg, animals with skin diseases or functions not relevant to humans such as fish or amphibians) ^4^Conference abstracts or preprints where a full paper has subsequently been published and was identified in the searches. ^5^Conference abstracts with insufficient information to extract (eg, for systematic reviews), duplicated reports (with differing doi), corrections to included reports.

With electronic database searching we identified 17 523 reports, which were uploaded to Covidence; from these, 8577 duplicates were removed. We excluded 17 933 reports based on dual screening of titles and abstracts. Additional searches identified a further 34 reports, resulting in a total of 1040 full texts, which were assessed for eligibility by 2 independent reviewers (7 full texts could not be retrieved). Of these, 523 reports were included in the review (Figure 1). Of the 523 reports included, 516 represented unique studies. The remaining 7 were secondary reports from the same studies and were excluded from study-level counts to prevent double-counting.

Data, including citation details for all included sources of evidence, is freely available without restriction (Supplementary Materials 3, Dataset). The majority (91%) of the included sources of evidence were primary research reports, while the remainder (6%) consisted mainly of conference abstracts. Conference abstracts were included only if not superseded by a primary research article, to capture emerging evidence in the area. Of the 523 included reports, it was established that 73 were systematic reviews, 22 were follow-up studies to an included study, and 7 were additional reports of an included study that provided no further information. Table 1 describes the 516 included studies (73 reviews and 443 experimental and observational studies) across the key categories of study type, participants, intervention/exposure, and outcome.

**Table 1.** Summary Characteristics of Included Studies That Investigated the Effects of Oral Probiotics, Prebiotics or Synbiotics on 1 or More Indicators of Skin Health, Function or Disease

Study characteristics (*n*)	Experimental/observational		Reviews	Total
**Type of evidence (*n* = 516)**				
Primary research article	401		70	471
Conference abstract	31		0	31
Preprint	0		1	1
Thesis	0		0	0
Research letters	6		1	7
Other	5		1	6
**Study types (*n* = 519^a^)**	**Human**	**Animal**		
Systematic review			73	73
Experimental—RCT	188	79		267
Experimental—Non-RCT	49	72		121
Observational—Cohort	17			17
Observational—Case-control	5			5
Observational—Cross-sectional	4			4
Observational—Case-series or case report	10			10
Follow-up of an included study	22			22
**Region where study conducted (*n* = 494^b^)**				
Asia	111	121	33	265
Europe	120	10	14	144
North America	16	13	17	46
South America	12	4	4	20
Oceania	9	1	5	15
Africa	2	0	0	2
Intercontinental	2	0	n/a	2
**Participant age (*n* = 770^c^)**				
Pregnant mothers	19	6	34	59
Infants (0-1 y)	114	6	57	177
Younger children (1-9 y)	83	2	32	117
Older children (10-19 y)	53	0	29	82
Adults (>18 y)	112	141	21	274
Older adults (>60 y)	41	5	15	61
**Participant characteristics (*n* = 497^d^)**				
Healthy	44	30	3	77
Specified skin type	15	1	1	17
Diagnosed with skin disease (self or clinician)	164	9	30	203
At risk population (eg, family history of skin disease)	25	0	20	45
Stimulated skin disease (animal studies)	n/a	108	0	108
Other nonskin disease/condition	25	3	2	30
Not specified			17	17
**Intervention/exposure (*n* = 529^e^)**				
Probiotic	206	127	68	401
Prebiotic	34	25	9	68
Synbiotic	41	6	13	60
**Skin outcome—prevention/management of disease (*n* = 427^f^)**				
Atopic dermatitis/eczema	179	87	64	330
Psoriasis	15	7	2	24
Acne	19	2	2	23
Urticaria	12	0	2	14
Skin cancer	1	12	0	13
Dandruff	4	0	0	4
Melasma	2	0	0	2
Alopecia	2	0	0	2
Rosacea	1	0	0	1
Non-specific inflammatory skin conditions	7	2	0	9
Other skin disease	3	2	0	5
*Any skin disease*	*245*	*112*	*70*	*427*
**Skin outcome—health/function (*n* = 140^g^)**				
Skin hydration/transepidermal water loss	23		2	
Wrinkling	13		0	
Elasticity	8		0	
UV irradiation response	6		0	
Wound healing	5		1	
Subjective skin condition	12		0	
Hair growth	1		0	
Sebum content	4		0	
Skin pH	2		0	
Dyspigmentation	2		0	
Skin barrier function	1		0	
Erythema	1		0	
Other	9		1	
*Any skin health/function*	*37*	*41* ^h^	*3*	*81*

a 3 studies report on 2 different experiment types resulting in 519 extracted interventions/exposures from 516 studies.

b 516 studies minus 22 follow-up studies.

c Total number of interventions/exposures per participant group, excluding follow-up studies; 29% of experimental/observational studies included participants from 2 age groups while 5% included participants from 3 or more age groups.

d 519 interventions/exposures minus 22 follow-up studies.

e Total number of interventions/exposures categories studied. For experimental/observational studies 9 interventions/exposures included 2 categories of probiotics, prebiotics or synbiotics and three interventions/exposures included all 3 categories.

f Total number of skin disease outcome categories studied; 9 experimental/observational studies measured 2 or more skin disease outcomes.

g Total number of skin health/function categories measured; 13 experimental/observational studies measured 2 skin health/function categories, while 14 studies measured three or more categories.

h Detailed breakout of outcomes not available for animal studies but the majority (61%) measured an outcome related to UV irradiation response and/or aging (wrinkling and/or elasticity), 17% wound healing, 12% hair growth and 10% skin hydration or Trans-Epidermal Water Loss.

### Study Characteristics

Although the earliest relevant studies were published in 1981 and 1991, it was only from 2006 onwards that more than 5 studies were published each year. Relevant publications have increased rapidly since 2006, with more than 40 studies published in each of 2022 and 2023. This rapid growth in publications, of which around 80% examine the use of oral probiotics, is illustrated in Supplementary Materials 3, Figure S1. The first systematic review was published in 2007, with reasonably steady growth in their publications since then, peaking at 10 publications in 2022 and 2023.

Experimental and observational studies in humans have been conducted primarily in Europe (*n* = 120, 44%), namely Italy (*n* = 26), Russia (*n* = 13), France (*n* = 11), Finland (*n* = 9), Ukraine (*n* = 8), Germany (*n* = 7), Netherlands (*n* = 7), Spain (*n* = 6), and the United Kingdom (*n* = 6); and Asia (*n* = 111, 41%), namely Japan (*n* = 29), China (*n* = 22), the Republic of Korea (*n* = 16), Iran (*n* = 16) and Taiwan (*n* = 6)). Thirteen (5%) human experimental/observational studies were conducted in the United States, 10 (4%) in Brazil, and 6 (2%) in Australia. Experimental data from animals originates primarily from Asia (*n* = 121, 81%), which is also the source of almost half (*n* = 33, 45%) of systematic reviews. These data are illustrated in Supplementary Materials 3, Figure S2.

### Participants

**Figure 2.**
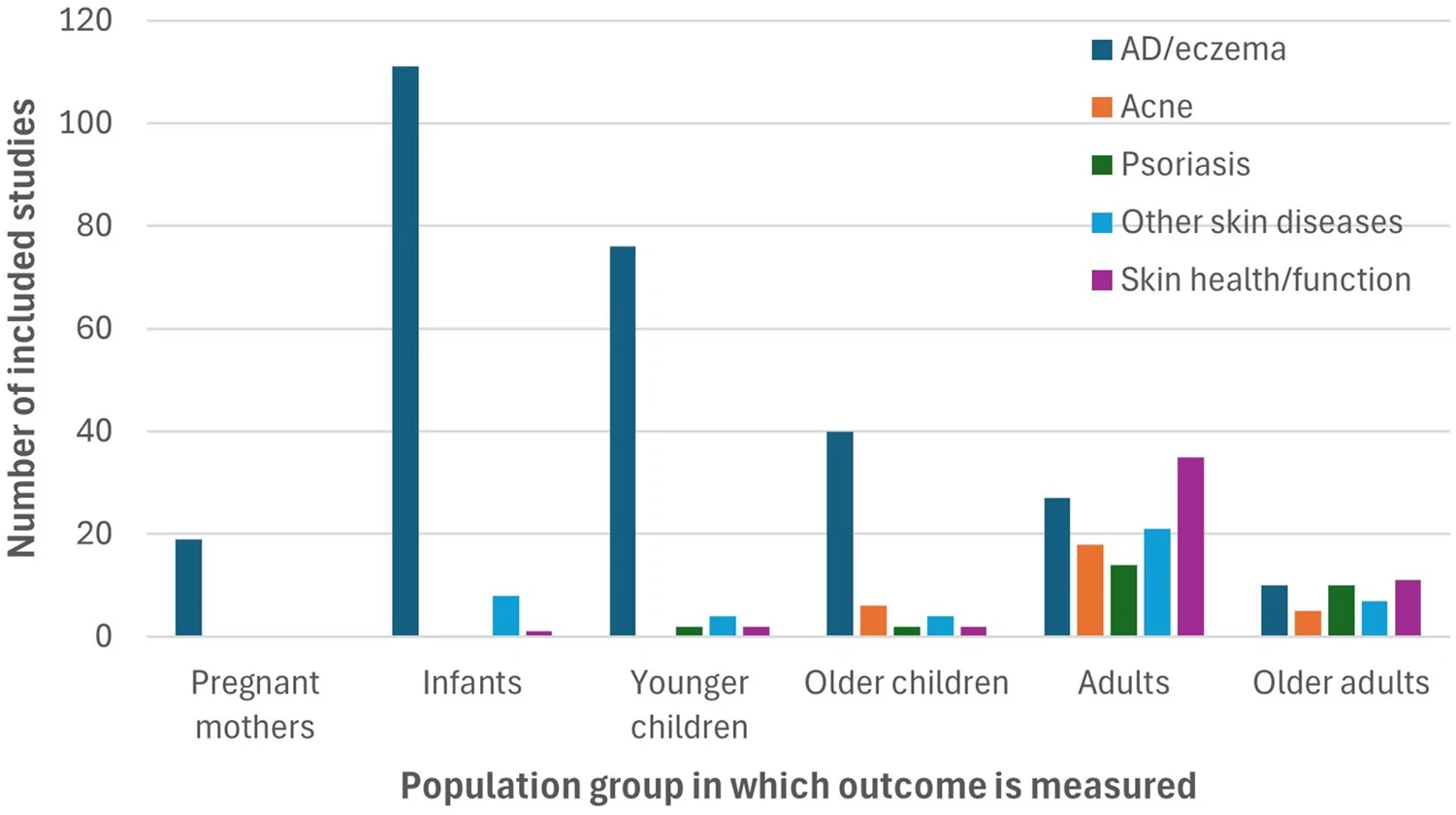
Numbers of Human Studies Investigating Oral Probiotics, Prebiotics, or Synbiotics and Skin Diseases or Skin Health/Function Outcomes Across the Life Course Data represent the number of studies of skin outcomes of the stated type conducted in the human population group shown. Abbreviation: AD, atopic dermatitis.

Infants were the most frequently studied human participant group, with 114 interventions/exposures (42%) assessing the use of supplements given before a child’s first birthday. Adults 18-60 years old were supplemented in 112 interventions/exposures (41%), while only 41 interventions/exposures (15%) were assessed in adults older than 60 years. Supplementary Materials 3, Figure S3 shows the frequency of each of probiotic, prebiotic, and synbiotic intervention/exposure in humans across the life course. The effects of oral probiotics, prebiotics, and synbiotics on skin disease prevention have been predominantly studied by supplementing pregnant mothers or infants (*n* = 67, 81% of studies measuring skin disease prevention), while their effect on skin health and function has been almost exclusively assessed in adults (*n* = 35, 69%) or older adults (*n* = 11, 22%) (see Supplementary Materials 3, Figure S4). Outcomes related to skin disease management have been studied more consistently across participant age groups; however, it is notable that 66% (*n* = 198) of the available evidence on disease management outcomes pertains to children 0-18 years old. The extent to which relevant skin outcomes have been examined across the life course is illustrated in Figure 2.

Just over a third (36%, *n* = 151) of experimental and observational studies used animal models rather than human participants. Of these animal experiments, 89% (*n* = 134) used mice, 6% (*n* = 9) dogs, and 5% (*n* = 8) rats. Different stages of the life course are less well represented in animal research; 93% (*n* = 141) of animal interventions were in adult animals, only 5% (*n* = 8) of interventions specified that infants or young pups were supplemented, 4% (*n* = 6) pregnant mothers and 3% (*n* = 5) older animals.

### Interventions/Exposures

#### Probiotics

**Figure 3.**
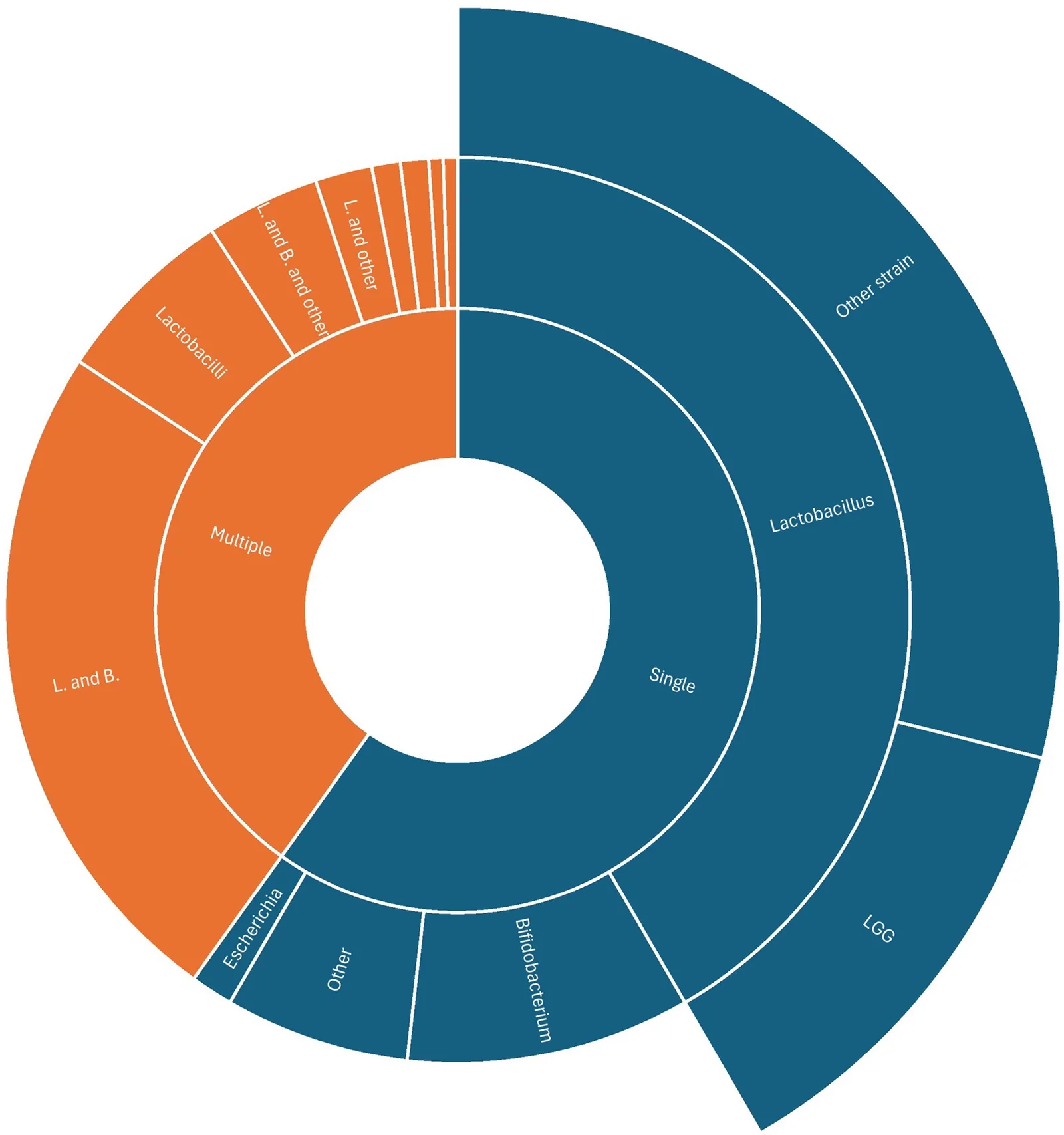
Breakdown of Probiotic Interventions/Exposures Studied Probiotic interventions or exposures (excluding synbiotics) reported in included studies investigating skin outcomes in humans are categorized based on the number of strains in the supplement, the genus of those strains, and where a single strain of *Lactobacillus* was administered whether that was the most frequently used strain, *Lactobacillus rhamnosus* GG, or another strain. Studies measuring both single and multiple strains, or strains across different genus categories are included in all categories that apply. Studies administering 2 strains of the same genus simultaneously are captured in the multiple strain category under the relevant genus. Abbreviations: L, *Lactobacillus*/i; B, *Bifidobacterium*/a; LGG, lactobacillus rhamnosus GG. 1. Probiotics have been categorized as described by the author with the exception of *Lacticaseibacillus casei*, *Lacticaseibacillus paracasei*, *Lacticaseibacillus rhamnosus*, *Lactiplantibacillus plantarum*, *Levilactobacillus brevis*, *Ligilactobacillus salivarius*, *Limosilactobacillus fermentum*, and *Limosilactobacillus reuteri*, which were renamed in 2020 and have been categorized as *Lactobacillus* for consistency with the majority of the research which was published prior to 2020.^59^ This affected 7 studies. 2. Foods fermented with *Streptococcus thermophilus* were excluded as they did not meet the probiotic definition; for this reason, this species was also excluded when categorizing multiple strains

Oral probiotic interventions/exposures were assessed in humans in 206 studies. Of these studies, 71% (*n* = 147) reported the strain(s) used in the supplement, and 70% (*n* = 144) reported the dose given in colony-forming units (CFUs) or equivalent. In comparison, only 18% (*n* = 37) reported that the strain(s) provided were known to reach the gut alive; 51% (*n* = 106) of interventions/exposures involved a single strain(s), 37% (*n* = 76) involved multiple strains, 2% (*n* = 4) both a single and multiple strain, and in 10% (*n* = 20) probiotic details were not provided. Figure 3 provides details of the genus of probiotics reported in 90% (*n* = 186) of these studies.

Although *Lactobacillus rhamnosus* GG was the most frequently used single-strain probiotic across the included studies, its effects on skin health have been predominantly studied in infants (*n* = 21) and younger children (*n* = 7), together accounting for 76% of the evidence on the effect of this strain on skin health (see Supplementary Materials 3, Figure S5).

#### Prebiotics

**Figure 4.**
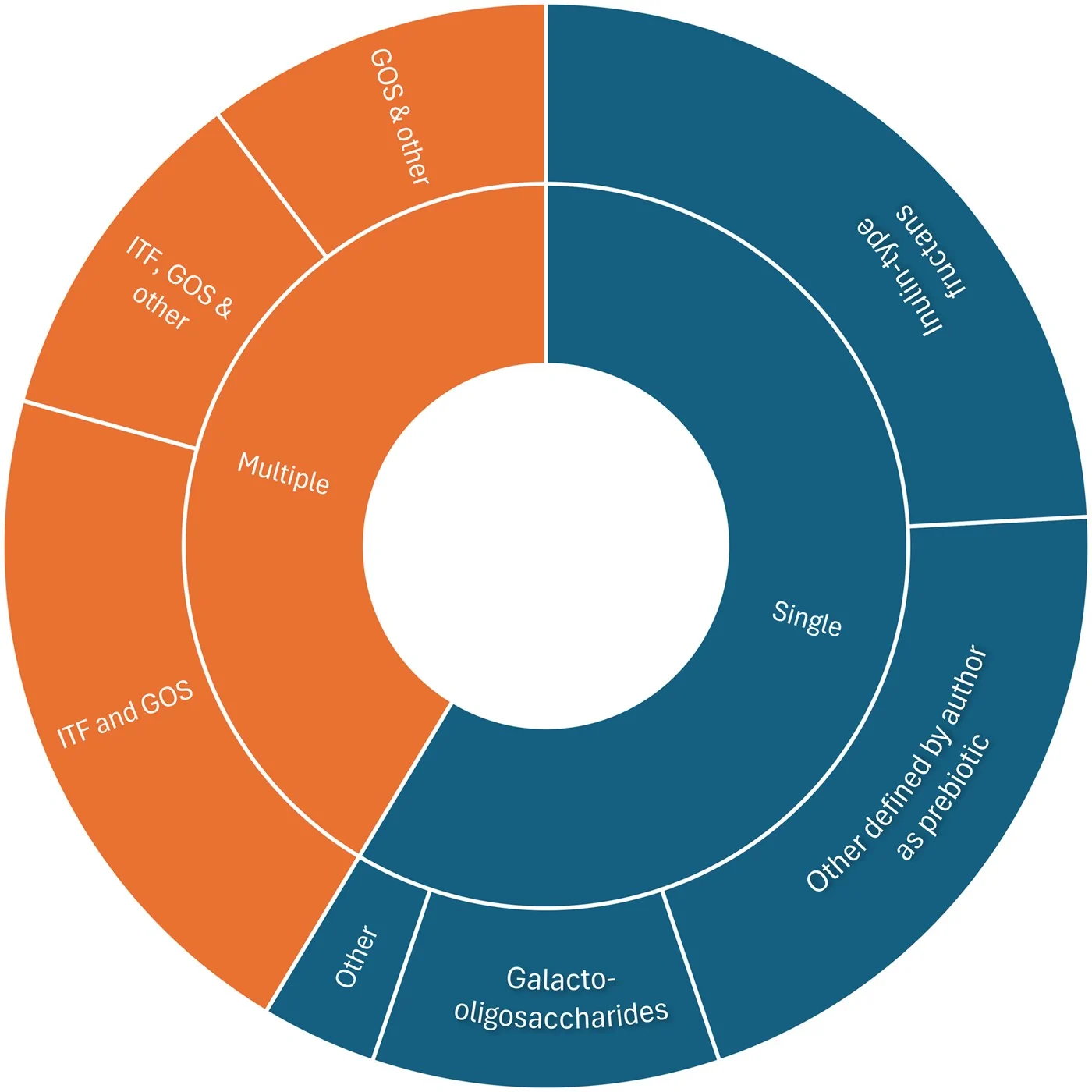
Breakdown of Prebiotic Interventions/Exposures Studied Prebiotic interventions or exposures (excluding synbiotics) reported in included studies investigating skin outcomes in humans are categorized based on the number of types of prebiotic in the supplement and the category of those prebiotics. Abbreviations: GOS, galactooligosaccharides; ITF, inulin-type fructans.

Oral prebiotic interventions/exposures were assessed in humans in 34 studies. The most frequently investigated categories of prebiotics were inulin-type fructans (ITFs) and galactooligosaccharides (GOSs), which were used in 47% (*n* = 16) and 44% (*n* = 15) of these interventions, respectively. These were used in combination in 26% of interventions or exposures (*n* = 9). Figure 4 provides details of the numbers and types of prebiotics supplied for 85% of these studies (*n* = 29) that reported such information.

#### Synbiotics

**Figure 5.**
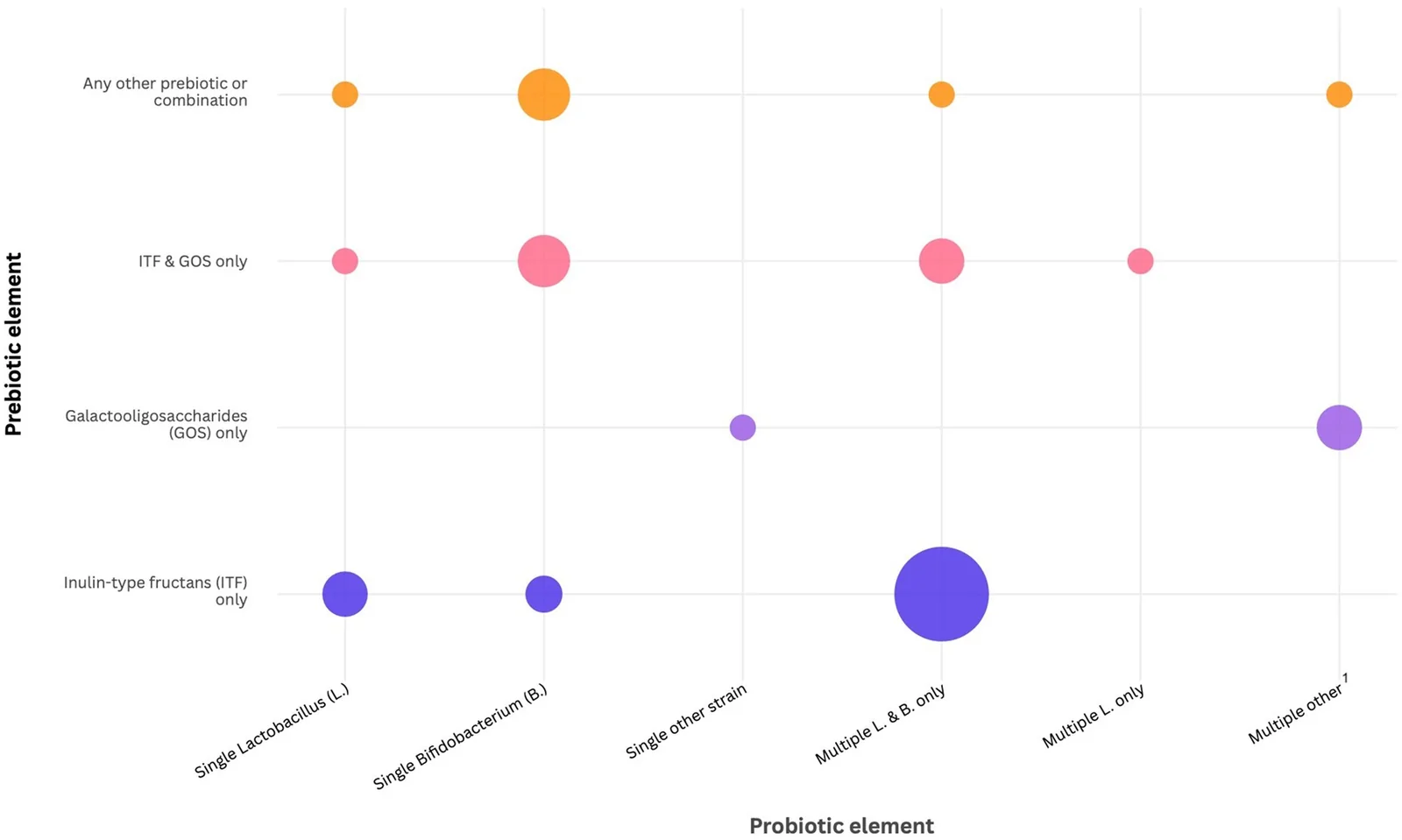
Breakdown of Synbiotic Interventions/Exposures Investigating Skin Outcomes in Humans. Size of bubbles represent number of synbiotic interventions using the category of probiotic and prebiotic elements shown. ^1^Foods fermented with *Streptococcus thermophilus* were excluded as they didn’t meet the probiotic definition so for this reason this species was also ignored when categorizing multiple strains

Oral synbiotic interventions/exposures were assessed in humans in 41 studies. Most commonly (in 34% of interventions/exposures, *n* = 13), a multiple-strain probiotic containing lactobacilli and bifidobacteria was combined with an ITF prebiotic. Figure 5 provides a breakdown of the frequency of synbiotic combinations, with the probiotic element shown on the *x*-axis and the prebiotic component on the *y*-axis.

#### Human Experimental Interventions

For interventions conducted in humans, further details on the characteristics of participants and the timing of exposures and outcome measurements are provided in Table 2. The median number of participants was 63, and 30% of trials (*n* = 71) had more than 100 participants. Females were well represented, accounting for 53% (*n* = 10 354) of the total participants in studies disclosing the sex split, and 13% of these studies (*n* = 24) were conducted in a female-only population. The majority (*n* = 149, 63%) of human interventions were conducted in populations previously diagnosed with a skin disease, while 14% (*n* = 34) of interventions were conducted in healthy populations. In 65% of studies (*n* = 154), supplementation with probiotics, prebiotics, and/or synbiotics was the sole intervention, while the remaining 35% (*n* = 83) used a co-intervention, most commonly an additional treatment (*n* = 38, 16%), such as topical treatments like emollients and/or steroids. The duration of interventions ranged from 1 week to 3 years, with a median of 12 weeks. Outcomes were typically measured during or immediately after exposure in 93% of studies (*n* = 220). In 24% of studies (*n* = 57) outcomes were measured after a further follow-up period, ranging from 1 week to 3 years, with a median of 22 weeks. Twenty-two of the included studies were additional follow-ups to an included human experimental study and are reported separately to avoid double-counting of intervention details. The follow-up periods of these studies were more extended, ranging from 18 months to 12.5 years.

**Table 2.** Intervention Details for Human Experimental Studies Investigating the Effects of Oral Probiotics, Prebiotics or Synbiotics on 1 or More Indicators of Skin Health, Function, or Disease

Human experimental study characteristics	Frequency, *n* (%)	
**Number of participants**		
0-20	17 (7%)	
21-50	76 (32%)	
51-100	73 (31%)	
101-500	66 (28%)	
>501	5 (2%)	
**Female participants, %**		
0% (males only)	3 (1%)	
1%-40%	39 (16%)	
40%-60%	84 (35%)	
60%-99%	38 (16%)	
100% (females only)	24 (10%)	
Not stated	49 (21%)	
**Participant characteristics**		
Healthy	34 (14%)	
Specified skin type^a^	15 (6%)	
Diagnosed with skin disease (self or clinician)	149 (63%)	
At-risk population (eg, family history of skin disease)	24 (10%)	
Other nonskin disease/condition^b^	15 (6%)	
**Other information reported**		
Ethnicity or skin type of participants	44 (19%)	
Dietary intake of participants was reported	19 (8%)	
Adjustments made for dietary intake of participants	2 (1%)	
Measurement of gut microbiome modulation^c^	66 (28%)	
**Co-interventions**		
Additional oral supplement	19 (8%)	
Additional treatment	38 (16%)	
Other intervention	11 (5%)	
Multiple/combination	15 (6%)	
None	154 (65%)	
**Duration of exposure**		
0-6 wk	59 (25%)	
6-12 wk	105 (44%)	
12-26 wk	49 (21%)	
6-12 mo	14 (6%)	
1-2 y	4 (2%)	
Variable or not stated	6 (3%)	
**Timing of initial outcome measurement**		
During or immediately after exposure	220 93%	
> 1 week after exposure	13 5%	
Variable or not stated	4 2%	
**Timing of final outcome measurement**	**Original studies**	**Follow-up studies**
No further follow-up measurement	180 (76%)	n/a
0-12 weeks	27 (11%)	0 (0%)
12-26 weeks	15 (6%)	0 (0%)
6-12 months	6 (3%)	0 (0%)
1-3 y	9 (4%)	6 (30%)
3-6 y	0 (0%)	8 (40%)
6-9 y	0 (0%)	4 (20%)
>9 y	0 (0%)	2 (10%)

a Examples include populations selected on the basis of dry or oily skin, burn patients or those with photodamaged skin.

b Examples include populations selected on the basis of an allergy, pre-term infants, diabetes or PCOS.

c Only if reported in the included study only, not captured if reported in a follow-up or additional report that wasn’t included in this review.

#### Outcomes

**Figure 6.**
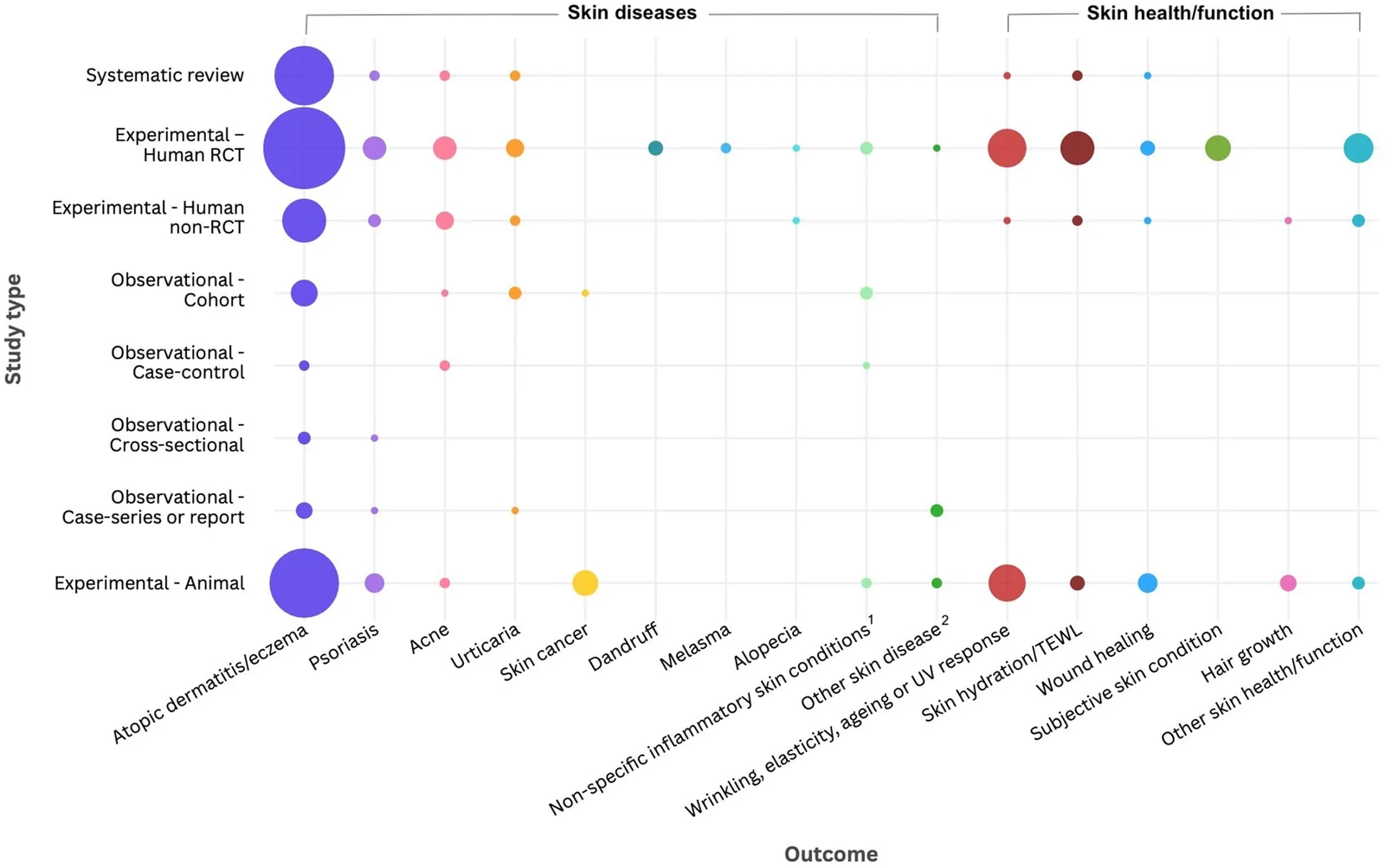
Map of Outcomes Studied Map of outcome categories by study type. Size of bubbles represent number of studies of oral probiotics, prebiotics or synbiotics measuring the outcomes shown using the study type shown. The studies counted in original research study categories (randomized controlled trials [RCTs], observational studies and animal experiments) are not mutually exclusive to the studies included in the systematic reviews. ^1^Non-specific inflammatory skin conditions include non-specified cutaneous symptoms/lesions, itchy rash/itching, papular-pustular exanthema and TPA-induced inflammation in mice ^2^Other skin diseases included rosacea, acute generalized exanthematous pustulosis, ichthyosis vulgaris, polymorphic light eruption and ulcers

Outcomes related to the prevention or management of atopic dermatitis account for the majority (*n* = 266, 54%) of those mapped in this review, across both experimental and observational studies, and an even greater majority (*n* = 64, 86%) of those mapped across systematic reviews. The second most widely reported outcome in experimental and observational studies was skin health and function as it relates to wrinkling, elasticity, aging, or UV irradiation response (*n* = 53, 11%), although almost half of this evidence comes from animal experiments. Figure 6 maps outcomes reported by study type, with the *y*-axis ordered according to the hierarchy of evidence.

For human experimental and observational studies, the subsequent figures illustrate the percentage and number of studies reporting a favorable effect or association of probiotics on the prevention or management of skin disease (Supplementary Materials 3, Figure S6) and skin health and function (Supplementary Materials 3, Figure S7), respectively. Note that all human study types are included, ranging from case reports to randomized controlled trials (RCTs), and studies were not rated for quality of evidence.

For more details on the links between the intervention/exposure categories of probiotics, prebiotics, and synbiotics and the various skin outcomes, see Supplementary Materials 3, Figure S8.

### Systematic Reviews

In some topic areas, studies were numerous enough to be systematically reviewed. Outcomes related to preventing and managing skin conditions were studied in 96% of reviews; only 3 reviews examined optimizing skin health and function (namely, skin hydration, epidermal thickness, and wound healing) in healthy populations. Atopic dermatitis was the most studied skin disease; 33 of 36 meta-analyses indicated a positive effect on its prevention, compared to 19 of 26 meta-analyses on atopic dermatitis severity and 1 of 6 meta-analyses on skin-related quality of life. The remaining reviews examined psoriasis (*n* = 2), urticaria (*n* = 2), and acne (*n* = 2).

A summary of the findings, along with conclusions and insights, is presented in Table 3.

**Table 3.** Summary of Key Findings and Corresponding Recommendations for Future Research

Area	Evidence availability	Key finding	Recommendations
AD	High	Majority of evidence relates to AD; 330 studies including 64 systematic reviews. Heterogeneity reported in evidence synthesis	Umbrella review suggested to evaluate consistency (4 currently registered ongoing)
Skin aging, wrinkling elasticity and UV response	Some	Growing area of research with 27 RCT outcomes and one systematic review (of 2 human RCTs)	Potential for systematic review to analyze efficacy and consider strains and doses
Psoriasis	Some	24 studies available (including 2 reviews, with the most recent finding positive effect on severity). No mention in dermatology clinical guidelines	Updated meta-analysis may be appropriate given recently published RCTs to confirm efficacy and facilitate possible translation into guidelines
Acne	Some	23 studies available (including 2 reviews, but neither with a meta-analysis). No mention in dermatology clinical guidelines	Potential for meta-analysis to analyze efficacy/strains/doses and facilitate possible translation into guidelines (2 registered as ongoing)
Skin cancer	Limited	Majority of limited evidence in animals—12 animal studies and 1 human observational cohort	Potential for further RCTs in humans to elucidate potential efficacy
Other skin diseases	Limited	Limited but initially encouraging evidence available on alopecia, rosacea, melasma, dandruff	Potential for further RCTs in humans to elucidate potential efficacy
Other skin health and function	Limited	Some evidence on skin hydration/TEWL and subjective condition with mixed outcomes. Limited evidence in other areas of skin health/function	Potential opportunity for industry innovation in developing and rigorously testing products
General		Inconsistent reporting of probiotic strains and doses. Heterogeneity reported in evidence synthesis	Design and report RCTs using both CONSORT and probiotic and prebiotic specific best practice guidelines
		Less than 10% of human intervention studies reported on participant diets	
		Poor reporting of ethnicity/skin type and representation of populations outside Europe and Asia	Ensure inclusive populations and no language limitations to facilitate generalizability and reduce bias
		Language limitations applied in 44% of reviews	

Abbreviations: AD, atopic dermatitis; RCT, randomized controlled trial; TEWL, trans-epidermal water loss.

## DISCUSSION

In this scoping review we have mapped and described the evidence available on oral probiotics, prebiotics, and synbiotics for skin health, function, and disease, categorizing 523 included sources of evidence by type of intervention or exposure investigated, outcomes across both skin health and function, and prevention and management of skin diseases, as well as life stages. While already extensive, this area of research appears to be rapidly expanding; half of the included studies have been published since 2018, and numerous trials and evidence syntheses are also ongoing.

### Types of Oral Probiotics, Prebiotics, and Synbiotics Investigated

The bulk of the evidence mapped relates to probiotics, with 79% experimental and observational studies and 93% reviews, analyzing supplementation with oral probiotics. Among the probiotics, the *Lactobacillus* genus has been the most widely studied to date, with 1 or more strains of *Lactobacillus* administered in 79% of probiotic experiments and observations, most often as a single strain (42% of studies). Evidence on multiple-strain probiotics is also widespread, accounting for 40% of studies. Combinations including 1 or more strains of both *Lactobacillus* and *Bifidobacterium* are the most common (28% of studies). This appears to be broadly consistent with research into the use of probiotics for other aspects of health beyond the skin, for example 78% of all probiotic products available in the United States listed as having evidence available for a beneficial effect on adult health contain 1 or more strain of lactobacilli, most commonly a single strain (34%) or in combination with 1 or more strains of bifidobacteria (22%).^20^ The most widely studied strain in this scoping review, *L. rhamnosus* LGG, has also been extensively studied beyond skin health and has been identified as having beneficial effects on both antibiotic-associated diarrhea and *Clostridium difficile*–associated diarrhea, as well as traveler’s diarrhea, the prevention of *Helicobacter pylori*, and inflammatory bowel disease in adults.^20^ Indeed, all the more widely studied strains identified in this review (such as *Bifidobacterium lactis* BB-12 and *Lactobacillus reuteri* DSM 17938) are recognized for their health benefits beyond the skin; there do not appear to be any widely investigated strains that are specific to the field of dermatology.^20^

In relation to prebiotics, available evidence on the link with skin health has expanded considerably since the current definition was agreed upon by ISAPP in 2016, with 54% of the 59 experimental/observational studies published since 2019. While the majority of studies have investigated the recognized prebiotic categories of ITF and GOS, multiple authors have also examined several other food-derived compounds as prebiotics in the context of skin health, including pectin/pectin-derived oligosaccharides (typically added to infant formulas alongside ITF and GOS), polydextrose, and lactulose. Lactulose is identified as a prebiotic by ISAPP, while polydextrose is identified as a promising candidate.^21^ Other compounds studied in isolation as prebiotics included poly-γ-glutamic acid, sulfated xylorhamnoglucuronan-rich extract, partially hydrolyzed guar gum, triphala, psyllium, yeast mannan, and lycopene. Prebiotics appear to be of increasing interest in skin health and function (which account for 21% of human prebiotic outcomes compared to 13% for probiotics and 10% for synbiotics) and are more widely used in adults (44% of prebiotic participant outcomes vs 37% for probiotics and 27% for synbiotics).

Synbiotic supplements were investigated in 47 experimental/observational studies and 13 reviews. Multiple probiotic strains were used more frequently than single strains; 58% of experimental and observational studies on synbiotic mixtures investigated multiple strains, compared to 40% of studies on probiotics alone. Conversely, the majority of synbiotic mixtures studied (68%) included only a single prebiotic compound, which is slightly higher than the 59% of prebiotic studies that used a single type of prebiotic. Since 2020, the ISAPP definition of synbiotics has included synergistic synbiotics, which comprise either a live microorganism or a substrate that, when considered individually, do not meet the criteria for a probiotic or a prebiotic, respectively.^8^ However, there seemed to be limited utilization of these types of synbiotics in the evidence mapped so far, with all studies describing probiotic and prebiotic components that individually meet criteria. The most widely used prebiotic components were ITF and GOS, used in 71% and 34% of synbiotics, respectively. Other compounds defined by the authors (or widely recognized) as prebiotic included 2'-fucosyllactose, isomalto-oligosaccharides (IMO), xylooligosaccharides, pectin-derived acidic oligosaccharide, raffinose, and agave-derived fructans. Over 60% of human studies on synbiotics have been conducted in infants, typically analyzing the effect of supplemented infant formula on the prevention or management of atopic dermatitis.

### Skin Health and Function Outcomes Measured

The effects of oral probiotics, prebiotics, and synbiotics on the prevention and management of atopic dermatitis have been widely studied in 64 systematic reviews, 121 human RCTs, and 145 other experimental or observational studies. Synthesizing and evaluating the results of these studies is beyond the remit of this scoping review. Nevertheless, the proportion of experimental and observational studies reporting at least 1 positive outcome was higher in studies addressing the management or severity of atopic dermatitis (72%) than in those examining prevention or prevalence (54%). These figures should be interpreted with caution, given the combination of study types and the lack of a rating for the quality of evidence. For example, it is noteworthy that while a similar proportion of human studies on prevention and management were RCTs (37/54 for prevention compared to 84/126 for management), the average number of participants was significantly lower in RCTs on management (72) than in RCTs on prevention (313). The summary of the proportion of positive outcomes should not therefore be used to draw assumptions about the overall strength of the evidence; this observation is underscored by the findings of the meta-analyses mapped, which account for quality of evidence, weighting of sample sizes and effect sizes, of which 33 of 36 (92%) indicated a positive effect on atopic dermatitis prevention, compared to 19 of 26 meta-analyses (73%) analyzing atopic dermatitis severity.

Beyond atopic dermatitis, the next most widely studied skin diseases in the context of oral probiotics, prebiotics, and synbiotics, based on the number of studies excluding reviews, are psoriasis (*n* = 22), acne (*n* = 21), skin cancer (*n* = 13), and urticaria (*n* = 12). Of these, acne has been most widely studied in humans, including 10 human RCTs with an average of 92 participants, of which 9 identified at least 1 positive effect of probiotics or synbiotics on disease severity. Two systematic reviews have also been published in 2023^22^ and 2024,^23^ both of which included 4 of the 10 RCTs, (7 RCTs have been published since 2021), and neither of which included a meta-analysis. Four systematic reviews are currently registered on PROSPERO in this field, of which 2 are due to include meta-analyses.^24–27^

The link between psoriasis and oral probiotics, prebiotics, and synbiotics has been investigated in 7 animal studies, 2 observational studies (1 cross-sectional and 1 case series), and 13 human experimental studies, of which 10 were RCTs and of which 7 identified a positive effect of probiotics or synbiotics. Two systematic reviews have been published in 2021^28^ and 2024, capturing 3 and 5 RCTs, respectively, both including meta-analyses. The latter identified a positive effect on psoriasis severity but not quality of life.^29^

The effects of oral probiotics, prebiotics, and synbiotics on skin health and function in healthy populations appears to be an emerging area of interest, with 11 human experimental studies published since 2022. Outcomes related to skin hydration, transepidermal water loss, aging, wrinkling, elasticity, and UV irradiation response are the most investigated. Skin care is a burgeoning area in terms of both market and research growth. Fifteen trials were identified as ongoing that investigated the effects of probiotics on skin health or function, and oral probiotic supplements explicitly marketed for skin health are commercially available. There may be an opportunity for industry innovation in developing and rigorously testing products in this area. It will also be important for healthcare professionals to stay informed about developments in the evidence and be aware of the potential implications for other health outcomes in patients who take probiotic supplements for their skin. Little is currently known about the use of such supplements in dermatology patients. Although outside the scope of this review it is relevant to note that topical probiotic skincare products have been commercially available for over 100 years and have gained significant popularity recently, with a variety of products now available worldwide that are claimed to have skin health benefits ranging from anti-wrinkling to acne management.^30^

### Life Stages

Mapping of included studies across skin conditions and life stages broadly followed the expected patterns: most studies on atopic dermatitis were conducted in infants and children, acne was predominantly studied in older children and adults, and psoriasis was studied in adults and older adults.

This review suggests that older adults are the most underrepresented life stage in this area of research. Adults older than 60 years were included in only 15% of the human experimental and observational studies. All of these studies also included adults under the age of 60 years, rather than focusing on older adults, and 37% of them stated that the oldest participant or maximum age was 70 years or less, leaving only 6% of these studies to include adults over the age of 70 years. Only 2 animal studies (no human studies) were identified on pressure ulcers, 1 of the skin conditions suffered disproportionately by those older than 70 years. One ongoing clinical trial was identified investigating pressure ulcers in 40 adult intensive care unit patients in Iran.^31^ The rapidly aging global population (the proportion more than 60 years old is expected to increase from 12% in 2015 to 22% by 2050^32^) and the fact that skin aging and its consequences (reduced healthy aging due to dry skin, chronic wounds and tumors as well as the impact on appearance) have been identified as a global challenge in skin health appears to present an opportunity for more research in this population group.

### Current Policy and Practice

Certain oral probiotics, prebiotics, and synbiotics are widely recognized for their gastroenterological health benefits, and the World Gastroenterological Organization has recently updated its clinical guidelines to recommend their use for various conditions.^33^ However, this is far from the case with dermatological conditions. Although a comprehensive review of dermatological clinical practice guidelines is beyond the scope of this review, it appears that the World Allergy Organization (WAO) is the only widely recognized organization to recommend the use of oral probiotics in relation to skin health, namely the prevention of atopic dermatitis in infants at high risk of allergic disease and their pregnant or lactating mothers.^34^ Notably, this is a conditional recommendation based on low-quality evidence. Developments in the evidence available for acne have not yet been reflected in policy guidelines. Although the 2016 version of the American Academy of Dermatology acne vulgaris management guidelines mentioned that the existing evidence on probiotics was insufficient to support any recommendations at that time, the recently updated guidelines do not mention them at all.^35^^,^^36^ Furthermore, probiotics are not mentioned i?n the United Kingdom National Institute for Health and Care Excellence (NICE) guidelines for the management of acne vulgaris.^37^

### Recommendations for Further Research

One of the objectives of this review was to identify gaps in the literature and make recommendations for research priorities. Regarding atopic dermatitis, the 64 systematic reviews available suggest the potential for an umbrella review to evaluate the consistency of the evidence base and investigate any discrepancies.^38^ Between conducting the searches for this review and November 2024, 3 further umbrella reviews of meta-analyses on probiotics, prebiotics, and/or synbiotics and atopic dermatitis have been registered on PROSPERO,^27^^,^^39^^,^^40^ to add to the 1 review registered in 2022, which has not yet been published.^41^ It is hoped that these reviews will bring some clarity to the synthesis of the large amount of information in this area, and perhaps facilitate translation into clinical guidelines where appropriate.

The number of systematic reviews on skin health/function is limited; outcomes related to wrinkling, elasticity, aging, or UV irradiation response account for 11% of the included experimental/observational studies, but there is only 1 systematic review in this area looking at photoaging, published in 2022 and including only 2 human RCTs,^42^ compared to the 27 RCT outcomes now available in these areas. This situation suggests that there may be scope for evidence synthesis in these areas to analyze the effectiveness of these supplements and help identify appropriate strains and doses.

There are several areas with numerous animal experiments, but evidence of effects in humans is more limited. The effects of oral probiotics, prebiotics, and synbiotics on skin cancer have been investigated in 12 animal studies, as well as 1 human study, an observational cohort study in patients with melanoma.^43^ Two clinical trials have been registered and appear to be ongoing, both looking at the effect of prebiotics.^44^^,^^45^ Further well-designed RCTs in humans are warranted to elucidate the potentially beneficial effects of probiotics, including the appropriate strains and doses, as well as prebiotics, on the management of skin cancers.

There are also many skin diseases where there is very limited, but initially encouraging evidence, such as rosacea,^46^ alopecia,^47^^,^^48^ melasma,^49^^,^^50^ and dandruff.^51–53^ These represent additional avenues for further research to investigate potential effects.

### Limitations of Included Sources

This review identified several limitations in the included sources of evidence. First, ethnicity and/or skin type were poorly reported by only 18% of human experimental studies, so comprehensive categorization of evidence by ethnicity wasn’t possible. This is important as risk factors for skin diseases vary by skin type; for example, UV exposure is not as significant a risk factor for melanoma development in people of color compared with fair-skinned populations.^54^ There were also inequalities in geographical representation; 85% of human experimental and observational studies were conducted in Europe or Asia, with minimal evidence available from Africa or South America. Moreover, given that both dietary patterns and geographical differences can significantly modulate gut microbiota, and with recent evidence indicating that probiotic-induced shifts may be transient,^55^ these factors warrant further investigation to elucidate the long-term efficacy of such interventions fully. Also notable is that 44% of included reviews specified a language restriction on their searches or eligibility criteria, most commonly English. This scoping review identified and included non-English sources, so language restrictions in the reviews represent an additional potential source of bias.

Another important consideration is that systematic reviews suggest significant heterogeneity between studies, attributed to variability in strains, doses, and follow-up periods.^12^ However, there is somewhat inconsistent reporting of the details of interventions and exposures. For example, 29% of human intervention studies failed to specify the strain of probiotic being administered, and 30% did not specify the dose in CFUs or equivalent units. Diet may also play a role in explaining variability in responses to interventions and exposures between individuals; evidence demonstrates that diet is a factor in both probiotic and prebiotic outcomes.^56^ However, only 8% of human intervention studies reported the participant diets. It is suggested that future RCTs be designed using both the CONSORT (Consolidated Standards of Reporting Trials) and probiotic- and prebiotic-specific best practice guidelines to ensure the generation of valid scientific evidence and facilitate future evidence synthesis with reduced heterogeneity.^56–58^

### Strengths and Limitations of This Review

The strengths of this scoping review are its breadth and the comprehensive mapping of the evidence available on skin health and oral probiotics, prebiotics, and synbiotics, following best practice methodology for scoping reviews and including gray literature such as conference abstracts, preprints, and theses. Although the inclusion criteria for prebiotics included substances such as polyphenols or polyunsaturated fatty acids that could meet ISAPP prebiotic criteria if they demonstrated an effect on bifidobacteria and/or *Lactobacillus* in the study, the search terms of this review did not include polyphenols or fatty acids. Evidence published before the 2017 and 2020 ISAPP definitions for prebiotics and synbiotics, respectively, which don’t use the terms prebiotic or synbiotic, may not have been captured by the searches if the effects on bifidobacteria and/or *Lactobacillus* were not mentioned in the abstract or keywords. When categorizing probiotic strains, this review relied on names provided by the authors of the included studies. Species that were no longer categorized within the genus *Lactobacillus* after 2020 were grouped as *Lactobacilli* for consistency with the vast majority of the data.^59^ This affected 7 included studies. Adverse effects were not captured unless they related to a skin outcome; full reporting on tests for safety and side effects would be essential to include in future systematic reviews and umbrella reviews. Finally, as this was a scoping review, no rating of the quality of evidence is provided, and therefore, implications for practice or policy cannot be graded.

## CONCLUSION

The key findings and resulting recommendations of this review are summarized in Table 3. Most of the evidence pertains to atopic dermatitis, for which an umbrella review would be appropriate. Research in other areas of dermatology, such as acne, psoriasis, and skin aging, remains limited; however, updated meta-analyses may help confirm efficacy and facilitate progress toward targeted dietary guidelines. General recommendations include improving study design and consistency of reporting to enable evidence synthesis, increasing inclusivity in participant demographics, and avoiding language limitations in systematic reviews to enhance generalizability.

This review has identified a wealth of evidence regarding oral probiotics, synbiotics, and atopic dermatitis. Given the numerous systematic reviews available, it is hoped that the registered umbrella reviews will bring increased clarity to evidence synthesis in this area. Further evidence synthesis may also be appropriate in the areas of acne and psoriasis to support potential translation into clinical guidelines, if warranted, as well as skin aging and UV irradiation response, which is an increasingly popular area of investigation. Less evidence is available for other skin diseases, such as skin cancer, alopecia, and rosacea, as well as areas of skin health and function beyond aging, offering potential for product innovation and trials to elucidate benefits in humans. It is recommended that future research be conducted in accordance with best practice guidelines to ensure the generation of valid scientific evidence and to minimize heterogeneity in future evidence synthesis.

## Supplementary Material

nuaf205_Supplementary_Data
