# Supplementary material for: Probiotics, Prebiotics, and Synbiotics as Oral Supplements for Skin Health, Function, and Disease Throughout the Life Course: A Scoping Review
Source: Nutr Rev. 2025 Nov 29;84(10):2174–92. doi: 10.1093/nutrit/nuaf205 (PMC13537454; doi:10.1093/nutrit/nuaf205)
Supplement: Supplementary file 1 [file nuaf205_supplementary_data.zip › Supplementary Materials R1.docx]

# Probiotics, prebiotics and synbiotics as oral supplements for skin health, function and disease throughout the life course: a scoping review

Rebecca A. Hillier

**Supplementary Materials**

**Supplementary Materials 1:** Database search details

**1. MEDLINE hosted on Ovid**

Ovid MEDLINE(R) ALL <1946 to March 08, 2024>

1	exp Prebiotics/	5165

2	exp Probiotics/	25564

3	exp Synbiotics/	1192

4	exp Oligosaccharides/ad, pd, tu [Administration & Dosage, Pharmacology, Therapeutic Use]	25373

5	exp Fructans/	8645

6	exp Chicory/	588

7	probiotic*.ti,ab,kf.	42208

8	prebiotic*.ti,ab,kf.	14255

9	synbiotic*.ti,ab,kf.	2711

10	inulin.ti,ab,kf.	9904

11	chicory.ti,ab,kf.	1072

12	fructan*.ti,ab,kf.	1864

13	fructo-oligosaccharide*.ti,ab,kf.	918

14	fructooligosaccharide*.ti,ab,kw.	1501

15	oligofructose.ti,ab,kf.	607

16	galactooligosaccharide*.ti,ab,kf.	701

17	galacto-oligosaccharide*.ti,ab,kf.	836

18	soya- oligosaccharide*.ti,ab,kf.	3

19	fermentable.ti,ab,kf.	5234

20	exp Bifidobacterium/	7482

21	exp Lactobacillaceae/	35310

22	Lactobacill*.ti,ab,kf.	49900

23	Bifidobac*.ti,ab,kf.	16029

24	Bacillus coagulans.ti,ab,kf.	779

25	Saccharomyces boulardii.ti,ab,kf.	936

26	Escherichia coli Nissle.ti,ab,kf.	397

27	Streptococcus thermophilus.ti,ab,kf.	2276

28	Streptococcus salivarius.ti,ab,kf.	1321

29	lactic acid bacter*.ti,ab,kf.	16157

30	exp Skin/	249615

31	exp Skin Diseases/	1250284

32	exp Skin Physiological Phenomena/	69545

33	skin.ti,ab,kf.	657936

34	epidermis.ti,ab,kf.	44498

35	dermat*.ti,ab,kf.	209938

36	dermal.ti,ab,kf.	65211

37	cutaneous.ti,ab,kf.	181652

38	facial.ti,ab,kf.	153472

39	acne.ti,ab,kf.	20105

40	psoriasis.ti,ab,kf.	52335

41	rosacea.ti,ab,kf.	4223

42	urticaria.ti,ab,kf.	17075

43	eczema.ti,ab,kf.	21061

44	pressure ulcer*.ti,ab,kf.	9357

45	erythema.ti,ab,kf.	35477

46	keloid.ti,ab,kf.	4325

47	telangiectasia.ti,ab,kf.	14223

48	photoaging.ti,ab,kf.	3333

49	Hidradenitis Suppurativa.ti,ab,kf.	4488

50	Dandruff.ti,ab,kf.	669

51	Pityriasis Versicolor.ti,ab,kf.	889

52	Folliculitis.ti,ab,kf.	2910

53	Alopecia areata.ti,ab,kf.	5407

54	Lupus.ti,ab,kf.	92971

55	Pemphig*.ti,ab,kf.	17134

56	Vitiligo.ti,ab,kf.	9174

57	Squamous cell carcinoma.ti,ab,kf.	116126

58	Basal cell carcinoma.ti,ab,kf.	14428

59	Melanoma.ti,ab,kf.	138836

60	Actinic Keratosis.ti,ab,kf.	3047

61	Merkel cell carcinoma.ti,ab,kf.	3884

62	(wound* adj3 heal*).ti,ab,kf.	105397

63	(wound* adj3 repair*).ti,ab,kf.	9987

64	exp Dietary Supplements/	104279

65	exp Administration, Oral/	158642

66	oral*.ti,ab,kf.	822846

67	supplement*.ti,ab,kf.	440200

68	dose*.ti,ab,kf.	1549114

69	tablet*.ti,ab,kf.	65340

70	powder*.ti,ab,kf.	100580

71	capsule*.ti,ab,kf.	94375

72	softgel*.ti,ab,kf.	146

73	food*.ti,ab,kf.	647667

74	beverage*.ti,ab,kf.	36937

75	drinking water.ti,ab,kf.	60030

76	ferment*.ti,ab,kf.	109533

77	mixture*.ti,ab,kf.	298214

78	or/1-29	149976

79	or/30-63	2342086

80	or/64-77	3734649

81	78 and 79 and 80	2889

**2. EMBASE hosted on Ovid**

Embase <1974 to 2024 March 08>

1	exp Prebiotics/	13468

2	exp Probiotics/	56170

3	exp Synbiotics/	3207

4	exp Oligosaccharides/ad, pd, tu [Administration & Dosage, Pharmacology, Therapeutic Use]	17325

5	exp Fructans/	1935

6	exp Chicory/	1438

7	probiotic*.ti,ab,kf.	51767

8	prebiotic*.ti,ab,kf.	16283

9	synbiotic*.ti,ab,kf.	3210

10	inulin.ti,ab,kf.	11118

11	chicory.ti,ab,kf.	1194

12	fructan*.ti,ab,kf.	2014

13	fructo-oligosaccharide*.ti,ab,kf.	1154

14	fructooligosaccharide*.ti,ab,kw.	1873

15	oligofructose.ti,ab,kf.	791

16	galactooligosaccharide*.ti,ab,kf.	894

17	galacto-oligosaccharide*.ti,ab,kf.	1014

18	soya- oligosaccharide*.ti,ab,kf.	4

19	fermentable.ti,ab,kf.	6025

20	exp Bifidobacterium/	23007

21	exp Lactobacillaceae/	68113

22	Lactobacill*.ti,ab,kf.	55817

23	Bifidobac*.ti,ab,kf.	20025

24	Bacillus coagulans.ti,ab,kf.	827

25	Saccharomyces boulardii.ti,ab,kf.	1285

26	Escherichia coli Nissle.ti,ab,kf.	490

27	Streptococcus thermophilus.ti,ab,kf.	2438

28	Streptococcus salivarius.ti,ab,kf.	1458

29	lactic acid bacter*.ti,ab,kf.	16766

30	exp Skin/	433253

31	exp Skin Diseases/	1847299

32	exp Skin Physiological Phenomena/	179209

33	skin.ti,ab,kf.	879325

34	epidermis.ti,ab,kf.	57447

35	dermat*.ti,ab,kf.	305549

36	dermal.ti,ab,kf.	87615

37	cutaneous.ti,ab,kf.	242633

38	facial.ti,ab,kf.	189352

39	acne.ti,ab,kf.	28686

40	psoriasis.ti,ab,kf.	79175

41	rosacea.ti,ab,kf.	5787

42	urticaria.ti,ab,kf.	27401

43	eczema.ti,ab,kf.	29982

44	pressure ulcer*.ti,ab,kf.	12155

45	erythema.ti,ab,kf.	54067

46	keloid.ti,ab,kf.	5390

47	telangiectasia.ti,ab,kf.	18838

48	photoaging.ti,ab,kf.	4474

49	Hidradenitis Suppurativa.ti,ab,kf.	6727

50	Dandruff.ti,ab,kf.	1131

51	Pityriasis Versicolor.ti,ab,kf.	1270

52	Folliculitis.ti,ab,kf.	4037

53	Alopecia areata.ti,ab,kf.	7157

54	Lupus.ti,ab,kf.	133309

55	Pemphig*.ti,ab,kf.	20237

56	Vitiligo.ti,ab,kf.	13160

57	Squamous cell carcinoma.ti,ab,kf.	161080

58	Basal cell carcinoma.ti,ab,kf.	20093

59	Melanoma.ti,ab,kf.	196213

60	Actinic Keratosis.ti,ab,kf.	4538

61	Merkel cell carcinoma.ti,ab,kf.	5761

62	(wound* adj3 heal*).ti,ab,kf.	137443

63	(wound* adj3 repair*).ti,ab,kf.	12683

64	exp Dietary Supplements/	24976

65	exp Administration, Oral/	401770

66	oral*.ti,ab,kf.	1141429

67	supplement*.ti,ab,kf.	556055

68	dose*.ti,ab,kf.	2251811

69	tablet*.ti,ab,kf.	109107

70	powder*.ti,ab,kf.	116387

71	capsule*.ti,ab,kf.	127670

72	softgel*.ti,ab,kf.	303

73	food*.ti,ab,kf.	769842

74	beverage*.ti,ab,kf.	45035

75	drinking water.ti,ab,kf.	78009

76	ferment*.ti,ab,kf.	120761

77	mixture*.ti,ab,kf.	342834

78	or/1-29	173684

79	or/30-63	3111099

80	or/64-77	5109417

81	78 and 79 and 80	4370

**3. CENTRAL The Cochrane Library hosted by Wiley**

Date Run:	11/03/2024 13:37:30

ID	Search	Hits

#1	MeSH descriptor: [Prebiotics] explode all trees	749

#2	MeSH descriptor: [Probiotics] explode all trees	3630

#3	MeSH descriptor: [Synbiotics] explode all trees	301

#4	MeSH descriptor: [Oligosaccharides] explode all trees and with qualifier(s): [pharmacology - PD, administration & dosage - AD, therapeutic use - TU]	2690

#5	MeSH descriptor: [Fructans] explode all trees	366

#6	MeSH descriptor: [Cichorium intybus] explode all trees	24

#7	(probiotic*):ti,ab,kw	10252

#8	(prebiotic*):ti,ab,kw	2181

#9	(synbiotic*):ti,ab,kw	1094

#10	(inulin):ti,ab,kw	1292

#11	(chicory):ti,ab,kw	129

#12	(fructan*):ti,ab,kw	233

#13	(fructo-oligosaccharide*):ti,ab,kw	311

#14	(fructooligosaccharide*):ti,ab,kw	617

#15	(oligofructose):ti,ab,kw	206

#16	(galactooligosaccharide*):ti,ab,kw	394

#17	(galacto-oligosaccharide*):ti,ab,kw	249

#18	(soya-oligosaccharide*):ti,ab,kw	1

#19	("fermentable"):ti,ab,kw	533

#20	MeSH descriptor: [Bifidobacterium] explode all trees	1062

#21	MeSH descriptor: [Lactobacillaceae] explode all trees	2228

#22	(Lactobacill*):ti,ab,kw	7026

#23	(Bifidobac*):ti,ab,kw	4310

#24	("Bacillus coagulans"):ti,ab,kw	190

#25	("Saccharomyces boulardii"):ti,ab,kw	363

#26	("Escherichia coli Nissle"):ti,ab,kw	37

#27	("Streptococcus thermophilus"):ti,ab,kw	366

#28	("Streptococcus salivarius"):ti,ab,kw	144

#29	(lactic acid bacter*):ti,ab,kw	796

#30	MeSH descriptor: [Skin] explode all trees	6138

#31	MeSH descriptor: [Skin Diseases] explode all trees	56064

#32	MeSH descriptor: [Skin Physiological Phenomena] explode all trees	4724

#33	(skin):ti,ab,kw	74039

#34	(epidermis):ti,ab,kw	1261

#35	(dermat*):ti,ab,kw	26515

#36	(dermal):ti,ab,kw	3167

#37	(cutaneous):ti,ab,kw	14808

#38	(facial):ti,ab,kw	13215

#39	(acne):ti,ab,kw	5570

#40	(psoriasis):ti,ab,kw	9927

#41	(rosacea):ti,ab,kw	747

#42	(urticaria):ti,ab,kw	3416

#43	(eczema):ti,ab,kw	5079

#44	(pressure NEXT ulcer*):ti,ab,kw	1968

#45	(erythema):ti,ab,kw	10220

#46	(keloid):ti,ab,kw	555

#47	(telangiectasia):ti,ab,kw	792

#48	(photoaging):ti,ab,kw	452

#49	("Hidradenitis Suppurativa"):ti,ab,kw	363

#50	(Dandruff):ti,ab,kw	245

#51	("Pityriasis Versicolor"):ti,ab,kw	151

#52	(Folliculitis):ti,ab,kw	319

#53	("Alopecia areata"):ti,ab,kw	801

#54	(Lupus):ti,ab,kw	4259

#55	(Pemphig*):ti,ab,kw	425

#56	(Vitiligo):ti,ab,kw	1258

#57	("Squamous cell carcinoma"):ti,ab,kw	7534

#58	("Basal cell carcinoma"):ti,ab,kw	1042

#59	(Melanoma):ti,ab,kw	6682

#60	("Actinic Keratosis"):ti,ab,kw	875

#61	("Merkel cell carcinoma"):ti,ab,kw	106

#62	(wound* NEAR/2 heal*):ti,ab,kw	14610

#63	(wound* NEAR/2 repair*):ti,ab,kw	344

#64	{OR #1-#29}	18986

#65	{OR #30-#63}	174590

#66	#64 AND #65	1410

#67	MeSH descriptor: [Dietary Supplements] explode all trees	19873

#68	MeSH descriptor: [Administration, Oral] explode all trees	29774

#69	(oral*):ti,ab,kw	228677

#70	(supplement*):ti,ab,kw	87519

#71	(dose*):ti,ab,kw	382669

#72	((oral* or mouth) NEAR/2 (administ* or treat*)):ti,ab,kw	80707

#73	(tablet*):ti,ab,kw	57629

#74	(powder*):ti,ab,kw	16763

#75	(capsule*):ti,ab,kw	38227

#76	(softgel*):ti,ab,kw	354

#77	(food*):ti,ab,kw	63032

#78	(beverage*):ti,ab,kw	8486

#79	("drinking water"):ti,ab,kw	1331

#80	(ferment*):ti,ab,kw	3413

#81	(mixture*):ti,ab,kw	12248

#82	{OR #67-#81}	662299

#83	#66 AND #82	1092

**4. CINAHL hosted by EBSCOhost**

Date run: 11 March 2024

| **#** | **Query** | **Limiters/Expanders** | **Last Run Via** | **Results** |
| --- | --- | --- | --- | --- |
| S78 | S73 AND S76 AND S77 | Expanders - Apply equivalent subjects  Search modes - Boolean/Phrase | Interface - EBSCOhost Research Databases  Search Screen - Advanced Search  Database - CINAHL | 843 |
| S77 | S61 OR S62 OR S63 OR S64 OR S65 OR S66 OR S67 OR S68 OR S69 OR S70 OR S71 OR S72 OR S74 OR S75 | Expanders - Apply equivalent subjects  Search modes - Boolean/Phrase | Interface - EBSCOhost Research Databases  Search Screen - Advanced Search  Database - CINAHL | 742,496 |
| S76 | S28 OR S29 OR S30 OR S31 OR S32 OR S33 OR S34 OR S35 OR S36 OR S37 OR S38 OR S39 OR S40 OR S41 OR S42 OR S43 OR S44 OR S45 OR S46 OR S47 OR S48 OR S49 OR S50 OR S51 OR S52 OR S53 OR S54 OR S55 OR S56 OR S57 OR S58 OR S59 OR S60 | Expanders - Apply equivalent subjects  Search modes - Boolean/Phrase | Interface - EBSCOhost Research Databases  Search Screen - Advanced Search  Database - CINAHL | Display |
| S75 | dose* | Expanders - Apply equivalent subjects  Search modes - Boolean/Phrase | Interface - EBSCOhost Research Databases  Search Screen - Advanced Search  Database - CINAHL | Display |
| S74 | supplement* | Expanders - Apply equivalent subjects  Search modes - Boolean/Phrase | Interface - EBSCOhost Research Databases  Search Screen - Advanced Search  Database - CINAHL | Display |
| S73 | S1 OR S2 OR S3 OR S4 OR S5 OR S6 OR S7 OR S8 OR S9 OR S10 OR S11 OR S12 OR S13 OR S14 OR S15 OR S16 OR S17 OR S18 OR S19 OR S20 OR S21 OR S22 OR S23 OR S24 OR S25 OR S26 OR S27 | Expanders - Apply equivalent subjects  Search modes - Boolean/Phrase | Interface - EBSCOhost Research Databases  Search Screen - Advanced Search  Database - CINAHL | Display |
| S72 | mixture* | Expanders - Apply equivalent subjects  Search modes - Boolean/Phrase | Interface - EBSCOhost Research Databases  Search Screen - Advanced Search  Database - CINAHL | Display |
| S71 | ferment* | Expanders - Apply equivalent subjects  Search modes - Boolean/Phrase | Interface - EBSCOhost Research Databases  Search Screen - Advanced Search  Database - CINAHL | Display |
| S70 | "drinking water" | Expanders - Apply equivalent subjects  Search modes - Boolean/Phrase | Interface - EBSCOhost Research Databases  Search Screen - Advanced Search  Database - CINAHL | Display |
| S69 | beverage* | Expanders - Apply equivalent subjects  Search modes - Boolean/Phrase | Interface - EBSCOhost Research Databases  Search Screen - Advanced Search  Database - CINAHL | Display |
| S68 | food* | Expanders - Apply equivalent subjects  Search modes - Boolean/Phrase | Interface - EBSCOhost Research Databases  Search Screen - Advanced Search  Database - CINAHL | Display |
| S67 | softgel* | Expanders - Apply equivalent subjects  Search modes - Boolean/Phrase | Interface - EBSCOhost Research Databases  Search Screen - Advanced Search  Database - CINAHL | Display |
| S66 | capsule* | Expanders - Apply equivalent subjects  Search modes - Boolean/Phrase | Interface - EBSCOhost Research Databases  Search Screen - Advanced Search  Database - CINAHL | Display |
| S65 | powder* | Expanders - Apply equivalent subjects  Search modes - Boolean/Phrase | Interface - EBSCOhost Research Databases  Search Screen - Advanced Search  Database - CINAHL | Display |
| S64 | tablet* | Expanders - Apply equivalent subjects  Search modes - Boolean/Phrase | Interface - EBSCOhost Research Databases  Search Screen - Advanced Search  Database - CINAHL | Display |
| S63 | oral* | Expanders - Apply equivalent subjects  Search modes - Boolean/Phrase | Interface - EBSCOhost Research Databases  Search Screen - Advanced Search  Database - CINAHL | Display |
| S62 | (MH "Administration, Oral+") | Expanders - Apply equivalent subjects  Search modes - Boolean/Phrase | Interface - EBSCOhost Research Databases  Search Screen - Advanced Search  Database - CINAHL | Display |
| S61 | (MH "Dietary Supplements+") | Expanders - Apply equivalent subjects  Search modes - Boolean/Phrase | Interface - EBSCOhost Research Databases  Search Screen - Advanced Search  Database - CINAHL | Display |
| S60 | wound* N2 repair* | Expanders - Apply equivalent subjects  Search modes - Boolean/Phrase | Interface - EBSCOhost Research Databases  Search Screen - Advanced Search  Database - CINAHL | Display |
| S59 | wound* N2 heal* | Expanders - Apply equivalent subjects  Search modes - Boolean/Phrase | Interface - EBSCOhost Research Databases  Search Screen - Advanced Search  Database - CINAHL | Display |
| S58 | "Merkel cell carcinoma" | Expanders - Apply equivalent subjects  Search modes - Boolean/Phrase | Interface - EBSCOhost Research Databases  Search Screen - Advanced Search  Database - CINAHL | Display |
| S57 | "Actinic Keratosis" | Expanders - Apply equivalent subjects  Search modes - Boolean/Phrase | Interface - EBSCOhost Research Databases  Search Screen - Advanced Search  Database - CINAHL | Display |
| S56 | Melanoma | Expanders - Apply equivalent subjects  Search modes - Boolean/Phrase | Interface - EBSCOhost Research Databases  Search Screen - Advanced Search  Database - CINAHL | Display |
| S55 | "Basal cell carcinoma" | Expanders - Apply equivalent subjects  Search modes - Boolean/Phrase | Interface - EBSCOhost Research Databases  Search Screen - Advanced Search  Database - CINAHL | Display |
| S54 | "Squamous cell carcinoma" | Expanders - Apply equivalent subjects  Search modes - Boolean/Phrase | Interface - EBSCOhost Research Databases  Search Screen - Advanced Search  Database - CINAHL | Display |
| S53 | Vitiligo | Expanders - Apply equivalent subjects  Search modes - Boolean/Phrase | Interface - EBSCOhost Research Databases  Search Screen - Advanced Search  Database - CINAHL | Display |
| S52 | Pemphig* | Expanders - Apply equivalent subjects  Search modes - Boolean/Phrase | Interface - EBSCOhost Research Databases  Search Screen - Advanced Search  Database - CINAHL | Display |
| S51 | Lupus | Expanders - Apply equivalent subjects  Search modes - Boolean/Phrase | Interface - EBSCOhost Research Databases  Search Screen - Advanced Search  Database - CINAHL | Display |
| S50 | "Alopecia areata" | Expanders - Apply equivalent subjects  Search modes - Boolean/Phrase | Interface - EBSCOhost Research Databases  Search Screen - Advanced Search  Database - CINAHL | Display |
| S49 | "Folliculitis" | Expanders - Apply equivalent subjects  Search modes - Boolean/Phrase | Interface - EBSCOhost Research Databases  Search Screen - Advanced Search  Database - CINAHL | Display |
| S48 | "Pityriasis Versicolor" | Expanders - Apply equivalent subjects  Search modes - Boolean/Phrase | Interface - EBSCOhost Research Databases  Search Screen - Advanced Search  Database - CINAHL | Display |
| S47 | Dandruff | Expanders - Apply equivalent subjects  Search modes - Boolean/Phrase | Interface - EBSCOhost Research Databases  Search Screen - Advanced Search  Database - CINAHL | Display |
| S46 | "Hidradenitis Suppurativa" | Expanders - Apply equivalent subjects  Search modes - Boolean/Phrase | Interface - EBSCOhost Research Databases  Search Screen - Advanced Search  Database - CINAHL | Display |
| S45 | photoaging | Expanders - Apply equivalent subjects  Search modes - Boolean/Phrase | Interface - EBSCOhost Research Databases  Search Screen - Advanced Search  Database - CINAHL | Display |
| S44 | telangiectasia | Expanders - Apply equivalent subjects  Search modes - Boolean/Phrase | Interface - EBSCOhost Research Databases  Search Screen - Advanced Search  Database - CINAHL | Display |
| S43 | keloid | Expanders - Apply equivalent subjects  Search modes - Boolean/Phrase | Interface - EBSCOhost Research Databases  Search Screen - Advanced Search  Database - CINAHL | Display |
| S42 | "erythema" | Expanders - Apply equivalent subjects  Search modes - Boolean/Phrase | Interface - EBSCOhost Research Databases  Search Screen - Advanced Search  Database - CINAHL | Display |
| S41 | pressure NEXT ulcer* | Expanders - Apply equivalent subjects  Search modes - Boolean/Phrase | Interface - EBSCOhost Research Databases  Search Screen - Advanced Search  Database - CINAHL | Display |
| S40 | eczema | Expanders - Apply equivalent subjects  Search modes - Boolean/Phrase | Interface - EBSCOhost Research Databases  Search Screen - Advanced Search  Database - CINAHL | Display |
| S39 | (MH "Urticaria") | Expanders - Apply equivalent subjects  Search modes - Boolean/Phrase | Interface - EBSCOhost Research Databases  Search Screen - Advanced Search  Database - CINAHL | Display |
| S38 | rosacea | Expanders - Apply equivalent subjects  Search modes - Boolean/Phrase | Interface - EBSCOhost Research Databases  Search Screen - Advanced Search  Database - CINAHL | Display |
| S37 | psoriasis | Expanders - Apply equivalent subjects  Search modes - Boolean/Phrase | Interface - EBSCOhost Research Databases  Search Screen - Advanced Search  Database - CINAHL | Display |
| S36 | "acne" | Expanders - Apply equivalent subjects  Search modes - Boolean/Phrase | Interface - EBSCOhost Research Databases  Search Screen - Advanced Search  Database - CINAHL | Display |
| S35 | facial | Expanders - Apply equivalent subjects  Search modes - Boolean/Phrase | Interface - EBSCOhost Research Databases  Search Screen - Advanced Search  Database - CINAHL | Display |
| S34 | cutaneous | Expanders - Apply equivalent subjects  Search modes - Boolean/Phrase | Interface - EBSCOhost Research Databases  Search Screen - Advanced Search  Database - CINAHL | Display |
| S33 | "dermal" | Expanders - Apply equivalent subjects  Search modes - Boolean/Phrase | Interface - EBSCOhost Research Databases  Search Screen - Advanced Search  Database - CINAHL | Display |
| S32 | "dermat*" | Expanders - Apply equivalent subjects  Search modes - Boolean/Phrase | Interface - EBSCOhost Research Databases  Search Screen - Advanced Search  Database - CINAHL | Display |
| S31 | epidermis | Expanders - Apply equivalent subjects  Search modes - Boolean/Phrase | Interface - EBSCOhost Research Databases  Search Screen - Advanced Search  Database - CINAHL | Display |
| S30 | skin | Expanders - Apply equivalent subjects  Search modes - Boolean/Phrase | Interface - EBSCOhost Research Databases  Search Screen - Advanced Search  Database - CINAHL | Display |
| S29 | (MH "Skin Diseases+") | Expanders - Apply equivalent subjects  Search modes - Boolean/Phrase | Interface - EBSCOhost Research Databases  Search Screen - Advanced Search  Database - CINAHL | Display |
| S28 | (MH "Skin+") | Expanders - Apply equivalent subjects  Search modes - Boolean/Phrase | Interface - EBSCOhost Research Databases  Search Screen - Advanced Search  Database - CINAHL | Display |
| S27 | "lactic acid bacter*" | Expanders - Apply equivalent subjects  Search modes - Boolean/Phrase | Interface - EBSCOhost Research Databases  Search Screen - Advanced Search  Database - CINAHL | Display |
| S26 | "Streptococcus salivarius" | Expanders - Apply equivalent subjects  Search modes - Boolean/Phrase | Interface - EBSCOhost Research Databases  Search Screen - Advanced Search  Database - CINAHL | Display |
| S25 | "Streptococcus thermophilus" | Expanders - Apply equivalent subjects  Search modes - Boolean/Phrase | Interface - EBSCOhost Research Databases  Search Screen - Advanced Search  Database - CINAHL | Display |
| S24 | "Escherichia coli Nissle" | Expanders - Apply equivalent subjects  Search modes - Boolean/Phrase | Interface - EBSCOhost Research Databases  Search Screen - Advanced Search  Database - CINAHL | Display |
| S23 | "Saccharomyces boulardii" | Expanders - Apply equivalent subjects  Search modes - Boolean/Phrase | Interface - EBSCOhost Research Databases  Search Screen - Advanced Search  Database - CINAHL | Display |
| S22 | "Bacillus coagulans" | Expanders - Apply equivalent subjects  Search modes - Boolean/Phrase | Interface - EBSCOhost Research Databases  Search Screen - Advanced Search  Database - CINAHL | Display |
| S21 | "Bifidobac*" | Expanders - Apply equivalent subjects  Search modes - Boolean/Phrase | Interface - EBSCOhost Research Databases  Search Screen - Advanced Search  Database - CINAHL | Display |
| S20 | "Lactobacill*" | Expanders - Apply equivalent subjects  Search modes - Boolean/Phrase | Interface - EBSCOhost Research Databases  Search Screen - Advanced Search  Database - CINAHL | Display |
| S19 | (MH "Lactobacillus+") | Expanders - Apply equivalent subjects  Search modes - Boolean/Phrase | Interface - EBSCOhost Research Databases  Search Screen - Advanced Search  Database - CINAHL | Display |
| S18 | (MH "Bifidobacterium+") | Expanders - Apply equivalent subjects  Search modes - Boolean/Phrase | Interface - EBSCOhost Research Databases  Search Screen - Advanced Search  Database - CINAHL | Display |
| S17 | "fermentable" | Expanders - Apply equivalent subjects  Search modes - Boolean/Phrase | Interface - EBSCOhost Research Databases  Search Screen - Advanced Search  Database - CINAHL | Display |
| S16 | ""soya-oligosaccharide*"" | Expanders - Apply equivalent subjects  Search modes - Boolean/Phrase | Interface - EBSCOhost Research Databases  Search Screen - Advanced Search  Database - CINAHL | Display |
| S15 | ""galacto-oligosaccharide*"" | Expanders - Apply equivalent subjects  Search modes - Boolean/Phrase | Interface - EBSCOhost Research Databases  Search Screen - Advanced Search  Database - CINAHL | Display |
| S14 | "galactooligosaccharide*" | Expanders - Apply equivalent subjects  Search modes - Boolean/Phrase | Interface - EBSCOhost Research Databases  Search Screen - Advanced Search  Database - CINAHL | Display |
| S13 | "oligofructose" | Expanders - Apply equivalent subjects  Search modes - Boolean/Phrase | Interface - EBSCOhost Research Databases  Search Screen - Advanced Search  Database - CINAHL | Display |
| S12 | "fructooligosaccharide*" | Expanders - Apply equivalent subjects  Search modes - Boolean/Phrase | Interface - EBSCOhost Research Databases  Search Screen - Advanced Search  Database - CINAHL | Display |
| S11 | "fructo-oligosaccharide*" | Expanders - Apply equivalent subjects  Search modes - Boolean/Phrase | Interface - EBSCOhost Research Databases  Search Screen - Advanced Search  Database - CINAHL | Display |
| S10 | "fructan*" | Expanders - Apply equivalent subjects  Search modes - Boolean/Phrase | Interface - EBSCOhost Research Databases  Search Screen - Advanced Search  Database - CINAHL | Display |
| S9 | "chicory" | Expanders - Apply equivalent subjects  Search modes - Boolean/Phrase | Interface - EBSCOhost Research Databases  Search Screen - Advanced Search  Database - CINAHL | Display |
| S8 | ""inulin"" | Expanders - Apply equivalent subjects  Search modes - Boolean/Phrase | Interface - EBSCOhost Research Databases  Search Screen - Advanced Search  Database - CINAHL | Display |
| S7 | "synbiotic*" | Expanders - Apply equivalent subjects  Search modes - Boolean/Phrase | Interface - EBSCOhost Research Databases  Search Screen - Advanced Search  Database - CINAHL | Display |
| S6 | "prebiotic*" | Expanders - Apply equivalent subjects  Search modes - Boolean/Phrase | Interface - EBSCOhost Research Databases  Search Screen - Advanced Search  Database - CINAHL | Display |
| S5 | "probiotic*" | Expanders - Apply equivalent subjects  Search modes - Boolean/Phrase | Interface - EBSCOhost Research Databases  Search Screen - Advanced Search  Database - CINAHL | Display |
| S4 | (MH "Oligosaccharides+/AD/PD/TU") | Expanders - Apply equivalent subjects  Search modes - Boolean/Phrase | Interface - EBSCOhost Research Databases  Search Screen - Advanced Search  Database - CINAHL | Display |
| S3 | (MH "Synbiotics+") | Expanders - Apply equivalent subjects  Search modes - Boolean/Phrase | Interface - EBSCOhost Research Databases  Search Screen - Advanced Search  Database - CINAHL | Display |
| S2 | (MH "Probiotics+") | Expanders - Apply equivalent subjects  Search modes - Boolean/Phrase | Interface - EBSCOhost Research Databases  Search Screen - Advanced Search  Database - CINAHL | Display |
| S1 | (MH "Prebiotics+") | Expanders - Apply equivalent subjects  Search modes - Boolean/Phrase | Interface - EBSCOhost Research Databases  Search Screen - Advanced Search  Database - CINAHL | Display |

**5. Scopus hosted by Clarivate**

Date run: 14 March 2024

Advanced query string used:

TITLE-ABS-KEY ( prebiotic* OR probiotic* OR synbiotic* OR fructan* OR chicory OR inulin OR fructo-oligosaccharide* OR fructooligosaccharide* OR oligofructose OR galactooligosaccharide* OR galactooligosaccharide* OR soya-oligosaccharide* OR fermentable OR lactobacill* OR bifidobac* OR "Bacillus coagulans" OR "Saccharomyces boulardii" OR "Escherichia coli Nissle" OR "Streptococcus thermophilus" OR "Streptococcus salivarius" OR "lactic acid bacter*" ) AND TITLE-ABS-KEY ( skin OR epidermis OR dermat* OR dermal OR cutaneous OR facial OR acne OR psoriasis OR rosacea OR urticaria OR eczema OR "pressure ulcer*" OR erythema OR keloid OR telangiectasia OR photoaging OR "Hidradenitis Suppurativa" OR dandruff OR "Pityriasis Versicolor" OR folliculitis OR "Alopecia areata" OR lupus OR pemphig* OR vitiligo OR "Squamous cell carcinoma" OR "Basal cell carcinoma" OR melanoma OR "Actinic Keratosis" OR "Merkel cell carcinoma" OR ( wound* W/2 heal* ) OR ( wound* W/2 repair* ) ) AND TITLE-ABS-KEY ( oral* OR supplement* OR dose* OR tablet* OR powder* OR capsule* OR softgel* OR food* OR beverage* OR "drinking water" OR "ferment*" OR mixture* )

**6. Web of science hosted by Elsevier**

\# Web of Science Search Strategy (v0.1)

\# Database: Web of Science Core Collection

\# Entitlements:

- WOS.SCI: 1900 to 2024

- WOS.AHCI: 1975 to 2024

- WOS.ESCI: 2015 to 2024

- WOS.ISTP: 1990 to 2024

- WOS.SSCI: 1956 to 2024

- WOS.ISSHP: 1990 to 2024

\# Searches:

1: TS=(prebiotic* OR probiotic* OR synbiotic* OR fructan* OR chicory OR inulin OR fructo-oligosaccharide* OR fructooligosaccharide* OR oligofructose OR galactooligosaccharide* OR galactooligosaccharide* OR soya-oligosaccharide* OR fermentable OR lactobacill* OR bifidobac* OR "Bacillus coagulans" OR "Saccharomyces boulardii" OR "Escherichia coli Nissle" OR "Streptococcus thermophilus" OR "Streptococcus salivarius" OR "lactic acid bacter*" )

Date Run: Wed Mar 13 2024 11:05:49 GMT+0000 (Greenwich Mean Time)

Results: 175415

2: TS=(skin OR epidermis OR dermat* OR dermal OR cutaneous OR facial OR acne OR psoriasis OR rosacea OR urticaria OR eczema OR "pressure ulcer*" OR erythema OR keloid OR telangiectasia OR photoaging OR "Hidradenitis Suppurativa" OR dandruff OR "Pityriasis Versicolor" OR folliculitis OR "Alopecia areata" OR lupus OR pemphig* OR vitiligo OR "Squamous cell carcinoma" OR "Basal cell carcinoma" OR melanoma OR "Actinic Keratosis" OR "Merkel cell carcinoma" OR ( wound* NEAR/2 heal* ) OR ( wound* NEAR/2 repair* ))

Date Run: Wed Mar 13 2024 11:06:33 GMT+0000 (Greenwich Mean Time)

Results: 1903808

3: TS=(oral* OR supplement* OR dose* OR tablet* OR powder* OR capsule* OR softgel* OR food* OR beverage* OR "drinking water" OR "ferment*" OR mixture* )

Date Run: Wed Mar 13 2024 11:06:59 GMT+0000 (Greenwich Mean Time)

Results: 5740511

4: #3 AND #2 AND #1

Date Run: Wed Mar 13 2024 11:07:24 GMT+0000 (Greenwich Mean Time)

Results: 3044

**Supplementary Materials 2:** Data extraction templates, with guidance

**Data extraction template for experimental and observational studies**

(continued on next page)

(continued on next page)

**Data extraction template for systematic reviews**

(continued on next page)

**Supplementary Materials 3:** Supplementary results

**Supplementary Dataset**

**Hillier R, Hall W. Details of studies included in scoping review: Probiotics, prebiotics and synbiotics as oral supplements for skin health, function and disease throughout the life course. Published online December 2024. doi:**[**10.18742/28044365.v1**](https://doi.org/10.18742/28044365.v1)

**Supplementary Figure 1:** **Year of publication of included experimental/observational studies**

Data represents cumulative number of studies available at the end of the year shown reporting on the intervention category shown. Studies reporting probiotic, prebiotic and synbiotic interventions or exposures are shown in blue, orange and grey respectively. 9 studies reported on two categories of probiotics, prebiotics or synbiotics so are counted twice, and three studies reported on all three categories so are counted three times. First included study was published in 1981.

**Supplementary Figure 2: Map of study types based on the continent in which the intervention/exposure was measured, or the review was authored**

Size of bubbles represent number of the study type shown.

**Supplementary Figure 3:** **Numbers of human studies investigating oral probiotics, prebiotics or synbiotics across the life course**

Number of includeed studies

| Population group in which intervention/exposure is studied | Probiotics | Prebiotics | Synbiotics |
| --- | --- | --- | --- |
| Pregnant mothers | 19 | 0 | 1 |
| Infants | 76 | 17 | 26 |
| Younger children | 61 | 7 | 16 |
| Older children | 45 | 4 | 5 |
| Adults | 87 | 14 | 13 |
| Older adults | 29 | 8 | 5 |

Data represents number of studies of interventions/exposures of the stated type conducted in the human population group shown. Studies reporting probiotic, prebiotic and synbiotic interventions or exposures are shown in blue, orange and grey respectively.

**Supplementary Figure 4**: **Numbers of human studies investigating skin disease prevention, management or skin health/function across the life course**

Number of includeed studies

| Population group in which outcome is measured | Disease prevention | Disease management | Skin health/function |
| --- | --- | --- | --- |
| Pregnant mothers | 19 | 0 | 0 |
| Infants | 48 | 71 | 1 |
| Younger children | 4 | 78 | 2 |
| Older children | 4 | 49 | 2 |
| Adults | 5 | 74 | 35 |
| Older adults | 3 | 28 | 11 |

Data represents number of studies of skin outcomes of the stated type conducted in the human population group shown. Studies reporting skin disease prevention, skin disease management and skin health and function outcomes are shown in blue, orange and grey respectively.

**Supplementary Figure** **5: Numbers of human studies investigating the effect of various strains of oral probiotics on skin health across the life course**

Number of includeed studies

| Population group in which outcome is measured | Multiple strains | Single - LGG | Single Lactobacillus (non-LGG) | Single bifidobacterium | Single other strain |
| --- | --- | --- | --- | --- | --- |
| Pregnant mothers | 11 | 3 | 4 | 1 | 0 |
| Infants | 24 | 21 | 16 | 11 | 6 |
| Younger children | 27 | 7 | 18 | 2 | 4 |
| Older children | 20 | 3 | 12 | 1 | 5 |
| Adults | 33 | 3 | 28 | 7 | 9 |
| Older adults | 11 | 0 | 8 | 4 | 2 |

Data represents number of studies supplementing with strains of the stated type conducted in the human population group shown.

**Supplementary Figure 6: Heatmap showing the frequency of a demonstrated beneficial effect of, or favourable association between, oral probiotics, prebiotics or synbiotics and skin disease outcome types**

**A:** shows percentage of studies reporting at least one positive outcome of the type shown. **B:** shows the total number of favourable (green), nil (orange) and negative (red) outcome types for each skin disease. * 1 study reports an outcome of this type; ** 2-10 studies report an outcome of this type; *** >10 studies report an outcome of this type

**Supplementary Figure 7: Heatmap showing the frequency of a demonstrated beneficial effect of, or favourable association between, oral probiotics, prebiotics or synbiotics and skin health and function outcomes**

**A:** shows the percentage of studies reporting at least one positive outcome. **B:** shows the total number of favourable (green), nil (orange) and negative (red) outcome types for each aspect of skin health or function on the right.

**Supplementary Figure 8**: **Number of human studies investigating the effect of oral probiotics, prebiotics and synbiotics on skin outcomes.**

|  | Probiotics | Prebiotics | Synbiotics |
| --- | --- | --- | --- |
| AD/eczema | 202 | 39 | 32 |
| Acne | 18 | 2 | 1 |
| Psoriasis | 18 | 1 | 3 |
| Other skin diseases | 40 | 5 | 4 |
| Skin health/function | 66 | 12 | 7 |

Data represents number of studies of interventions/exposures where the outcomes measured are in the categories shown. Studies reporting probiotic, prebiotic and synbiotic interventions or exposures are shown in blue, orange and grey respectively.
